# CoMuRoS - An LLM-based generalizable hierarchical task planning and execution framework for heterogeneous robot teams with event-driven re-planning

**DOI:** 10.3389/frobt.2026.1843313

**Published:** 2026-08-04

**Authors:** Suraj Borate, Bhavish Rai B, Vipul Pardeshi, Madhu Vadali

**Affiliations:** 1 Department of Mechanical Engineering, IIT Gandhinagar, Gandhinagar, Gujarat, India; 2 Computer Engineering, Vishwakarma Institute of Information Technology, Pune, Maharashtra, India

**Keywords:** event-driven re-planning, generalization, hierarchical task planning, human-robot collaboration, LLM, multi-robot systems, ROS2, vision-language models

## Abstract

Heterogeneous multi-robot teams require systems that can interpret natural-language goals, allocate tasks, and adapt to unexpected events. We developed CoMuRoS (Collaborative Multi-Robot System), a generalizable hierarchical architecture combining a centralized task-manager LLM with decentralized robot-level LLMs for executable Python generation from primitive ROS2 skills. The task manager uses static planning rules and dynamic context, including task history, robot/task status, and detected events, while onboard perception using VLM/image processing classifies events as relevant or irrelevant and triggers replanning. Hardware experiments demonstrated recovery from disruptive events, filtering of irrelevant distractions, and coordinated transport with emergent human-robot cooperation, achieving success rates of 9/10 for collaborative object recovery, 8/8 for coordinated transport, and 5/5 for human-assisted recovery. Simulation studies demonstrated intention-aware replanning. A curated benchmark of 22 scenarios, 54 tasks, and around 20 robots evaluated task allocation, classification, IoU, executability, and correctness across multiple LLMs, with correctness up to 0.91 ± 0.053; a 20-scenario replanning benchmark achieved Correctness = 0.948 ± 0.034 using Grok 3. CoMuRoS enables runtime, event-driven replanning on physical robots and supports flexible multi-robot and human-robot collaboration across diverse scenarios.

## 1 Introduction

Multi-robot systems have the potential to significantly improve efficiency, scalability, and robustness in complex real-world applications such as disaster response, logistics, and environmental monitoring (Karapetyan et al., 2017; Alonso-Mora et al., 2017). However, enabling effective cooperation among heterogeneous robots remains a fundamental challenge. In particular, real-world deployments require systems that can (i) interpret high-level human instructions, (ii) allocate tasks based on diverse robot capabilities, and (iii) adapt to dynamic environments where unexpected events can occur.

Existing multi-robot task allocation and planning approaches are often limited by rigid task representations, predefined dependency graphs, or strong assumptions about environment dynamics (Fikes and Nilsson, 1971; Aeronautiques et al., 1998; Wang et al., 2024; Li et al., 2026). Such methods struggle to handle open-ended human instructions, evolving task requirements, and unforeseen disruptions. While recent works have explored the use of large language models (LLMs) and vision-language models (VLMs) for high-level reasoning, most approaches either focus on single-robot systems or lack mechanisms for structured coordination and real-time re-planning in multi-robot settings.

Human teams naturally address these challenges. They can interpret natural language instructions, decompose tasks based on individual capabilities, and adapt plans dynamically in response to unexpected events through cooperation. Translating these capabilities to multi-robot systems is essential for enabling robust autonomy and effective human-robot interaction in dynamic environments.

Motivated by this, this paper addresses the following key research questions:

- **Q1:** How can a heterogeneous multi-robot system reliably interpret and execute high-level natural language instructions?
- **Q2:** How can robots cooperate and assist one another in response to events that disrupt ongoing task execution?
- **Q3:** How can a generalizable multi-robot architecture be designed to support operation across diverse environments, applications, robot morphologies, and task types?

To emulate the capabilities of human teams, it is intuitive to draw inspiration from the mechanisms through which humans plan, coordinate, and adapt in collaborative settings.

In this paper, we present *CoMuRoS*, a hierarchical systems architecture for zero-shot task understanding, structured task allocation, and event-driven re-planning in heterogeneous multi-robot teams. Here, “zero-shot” denotes that the same task manager prompt, defining coordination, teamwork, task allocation, task classification, and replanning rules, is applied across multiple scenarios without scenario specific fine tuning, retraining, or prompt modification. The proposed system is inspired by human organizational structures, where a central manager assigns tasks based on team capabilities, while individual agents execute tasks autonomously and report relevant events.

In CoMuRoS, a centralized *task manager LLM* interprets user intent expressed in natural language and decomposes it into executable sub-tasks. These tasks are allocated to robots based on their capabilities, morphologies, and constraints. Each robot then uses a local LLM to translate assigned tasks into executable actions using its available skill set. Crucially, the system incorporates an event-driven feedback mechanism, where robots proactively report relevant events (e.g., failures, environmental changes, or human interactions), triggering adaptive re-planning and task reallocation. This enables seamless integration of multi-robot coordination and human-in-the-loop collaboration in dynamic environments.

The effectiveness of CoMuRoS is demonstrated through both hardware experiments and large-scale simulations. Hardware experiments show that heterogeneous teams can autonomously recover from failures, ignore irrelevant disturbances, and perform coordinated manipulation tasks. Simulation studies further illustrate the system’s ability to generalize across diverse domains, including hospital assistance and disaster response scenarios. To systematically evaluate performance, we introduce a benchmark dataset consisting of 22 scenarios and 54 tasks involving approximately 20 robots, assessing task classification, allocation, planning correctness, executability, and robustness to variations in user instructions and LLM backends.

The *key contributions* of this work are:

1. A classification-guided hierarchical task management framework that structurally differentiates independent, sequential, and coordinated, enabling parallel dispatch, queue-based execution, and team-level coordination within a unified decision loop.
2. Hardware demonstrations of runtime event-driven re-planning using LLM-based task management in heterogeneous multi-robot teams, enabling controlled interruption, task reallocation, and continuation after unexpected events. The event-driven replanning results in emergent multi-robot cooperation.
3. A generalizable and flexible multi-robot systems framework that adapts to new scenarios and robot configurations using textual scenario configuration file without requiring strict manual dependency modeling. The architecture’s generalization is demonstrated through its task planning and replanning capabilities, validated across a custom textual scenario dataset as well as hardware and simulation experiments conducted in diverse scenario contexts.

## 2 Related work

LLM task planners (Huang et al., 2022) and robot foundation models (Shah et al., 2023; Firoozi et al., 2025) represent two distinct paradigms for robot task planning and execution. Robot foundation models or vision-language action models (VLAs), such as RT-1/RT-2 (Brohan et al., 2022; Zitkovich et al., 2023), OpenVLA (Kim et al., 2024), and GR00T N1 (Bjorck et al., 2025), directly map visual or sensor observations and natural language commands to robot actions. While effective for manipulation and single-robot scenarios, they are not designed for multi-robot systems. Their closed-loop policies are trained on demonstration datasets and fine-tuned for single robots, limiting performance on long-horizon tasks. They also struggle to reason about relevant events (e.g., failures or environment changes) and generalize less effectively than zero-shot LLM planners. In contrast, LLM-based planners use common-sense reasoning, integrate symbolic knowledge of robot capabilities, and deliberate over alternatives, offering stronger robustness in dynamic conditions. VLAs, imitation learning, reinforcement learning (RL) (Barto, 2021), and classical planning-control (Spong and Vidyasagar, 2008) remain valuable at the policy or primitive-action level but require a deliberative planner, such as an LLM, for long-horizon task planning and event-driven replanning.

Early planning frameworks like PDDL (Aeronautiques et al., 1998) or classical symbolic reasoning (Fikes and Nilsson, 1971) work reliably in fully known environments but lack adaptability in open-ended or uncertain domains. Recently, LLM-based planners have emerged as alternatives by combining atomic robot actions into valid sequences in unseen settings for multi-robot systems. SMART-LLM (Kannan et al., 2024), for example, demonstrated Python-based planning but lacked ROS2 integration, hierarchical design, or a dialogue interface, and did not test event-driven replanning. COHERENT (Liu et al., 2025) selects atomic actions from object locations but does not generate Python code, assumes full observability, and has not demonstrated event-driven replanning.

A central challenge in multi-robot settings is balancing centralized and decentralized planning. Frameworks like Decentralized Multi-Agent Systems (DMAS) (Zhang et al., 2023) and RoCo (Mandi et al., 2024) explored dialogue-based decentralized systems but suffer from token inefficiency and poor scalability as robot numbers grow (Chen et al., 2024). introduced Hybrid Multi-Agent Systems (HMAS-1/2), combining centralized and decentralized reasoning. HMAS-2 showed improved success rate and token efficiency in coordination tasks, though event-driven replanning in dynamic settings was not explored. This result motivates the hybrid hierarchical design of CoMuRoS: a centralized task manager for high-level deliberation combined with decentralized robot-level execution. Chen et al. (2024) demonstrated that such hybrid hierarchical architectures outperform both fully centralized and fully decentralized approaches in multi-robot coordination efficiency and scalability, providing a strong empirical basis for this architectural choice.

The CoMuRoS framework takes a hierarchical approach where the LLM acts as a high-level planner, using zero-shot reasoning to generate plans, classify tasks, and replan when events occur. Execution is delegated to robot modules handling low-level control via ROS2, classical controllers, or VLA/RL policies. This ensures the LLM focuses on deliberation while robot-level systems ensure reliable execution. Unlike prior work, CoMuRoS supports event-driven replanning, scalable ROS integration, and generalizable coordination across heterogeneous teams. A comparison of existing LLM-based multi-robot planners with CoMuRoS, focusing on framework type, replanning, human collaboration, hardware validation, dependency modeling, modularity, perception/event integration, and ROS2 support is given in Table 1.

**TABLE 1:** Comparison of LLM-based multi-robot systems.

Architecture name	CoELA	SMART-LLM	RoCo	COHER-ENT	DART-LLM	Env-LTL+LLM	CoMuRoS
Author(s) and Year	Zhang et al. (2023)	Kannan et al. (2024)	Mandi et al. (2024)	Liu et al. (2025)	Wang et al. (2024)	Li et al. (2026)	(Ours)
Multi-Robot Support	**✓**	**✓**	**✓**	**✓**	**✓**	**✓**	**✓**
Heterogeneous Robots	**✓**	**✓**	**✓**	**✓**	**✓**	**✓**	**✓**
Framework Type (Centralized/ Decentralized/ Hybrid)	Dec.	Cent	Dec.	Hybrid	Hybrid	Cent	Hybrid
Replanning Capability	**✓**	**✗**	**✓**	**✓**	**✓**	**✓**	**✓**
Event-Driven Replanning (Simulation)	**✗**	**✗**	**✗**	**✗**	**✗**	**✓**	**✓**
Human-Robot Collaboration	**✗**	**✗**	**✓**	**✗**	**✗**	**✗**	**✓**
Hardware Demonstration	**✗**	**✓**	**✓**	**✓**	**✓**	**✗**	**✓**
Event-Driven Replanning (Hardware)	**✗**	**✗**	**✗**	**✗**	**✗**	**✗**	**✓**
Explicit Dependency Modeling	**✗**	**✗**	**✗**	**✓**	**✓**	**✓**	Implicit
Modular Configuration (Generalization)	**✗**	**✗**	**✗**	**✗**	**✗**	**✗**	**✓**
VLM Integration (Perception/Events)	**✗**	**✗**	**✗**	**✗**	**✗**	**✗**	**✓**
Open-source ROS2 Framework	**✗**	**✗**	**✗**	**✗**	**✗**	**✗**	**✓**

Explicit dependency modeling, as in DART-LLM’s (Wang et al., 2024) Directed Acyclic Graph (DAG) or COHERENT’s (Liu et al., 2025) Proposal-Execution-Feedback-Adjustment (PEFA), enforces deterministic ordering and correctness in well-defined workflows. However, these methods are rigid when unexpected events or new agents appear, require heavy engineering to encode preconditions and effects, and usually assume near-complete observability. In contrast, CoMuRoS resolves dependencies implicitly through context-driven reasoning, task classification, task-status monitoring, and event feedback. Robots proactively report completions and interruptions, enabling the task manager to infer sequential dependencies, launch parallel tasks, or trigger replanning. This trades strict guarantees for adaptability, supporting event-driven replanning, seamless human interaction, generalization, and emergent multi-robot and human-multirobot collaboration. Li et al. (2026) proposed an environment-driven LTL framework that monitors predefined temporal-logic constraints and triggers replanning when symbolic environment specifications are violated, regenerating robot-level tasks accordingly. In contrast, CoMuRoS does not rely on a predefined task graph or formal constraint-violation model; instead, any runtime condition that impacts task allocation, such as new user instructions, human assistance, task disruptions, state changes, or newly introduced goals is treated as a relevant event. Thus, replanning in CoMuRoS is driven by open-world execution-level changes rather than solely by logical constraint violations. Systems using explicit dependency graphs (e.g., DART-LLM) or formal constraint models (e.g., COHERENT’s PEFA) offer stronger correctness guarantees in well-specified, static domains. CoMuRoS intentionally trades strict ordering guarantees for open-world adaptability, essential for event-driven replanning where the event and objects in the environment space cannot be strictly pre-enumerated. Neither DART-LLM nor COHERENT demonstrates event-driven replanning capability (Table 1), making a meaningful runtime comparison on this central contribution infeasible.

While RoCo (Mandi et al., 2024), COHERENT (Liu et al., 2025), DART-LLM (Wang et al., 2024), Environment Driven LTL and LLM based Framework (Li et al., 2026) and CoELA (Zhang et al., 2023) include some form of replanning, all remain limited and lack runtime recovery in hardware. RoCo uses a validation loop where infeasible subtasks (e.g., failing IK or collisions) are corrected before execution, but no mid-execution replanning occurs. COHERENT’s PEFA loop iteratively refines plans using robot feedback from completed tasks rather than unexpected events. DART-LLM represents tasks as a DAG, deferring or rescheduling subtasks when prerequisites fail, but it does not generate entirely new plans or reassign tasks to other robots. CoELA emphasizes decentralized planning and communication efficiency, but lacks event-driven replanning. By contrast to LLM based multi-robot task planning systems featured in Table 1, CoMuRoS demonstrates event-driven replanning on physical robots: failures such as object drops or human intent changes trigger the task manager to generate a new plan, reallocate tasks (including to other robots or humans), and continue execution. This dynamic recovery enables emergent cooperation in both multi-robot and human-robot settings, absent in prior work.

## 3 Methodology

This section presents the proposed CoMuRoS architecture for hierarchical task planning and execution in heterogeneous multi-robot systems. The framework is designed to address the key challenges of natural language task understanding, cooperative adaptation to disruptive events, and generalizable operation across diverse scenarios, as outlined in Section 1. In the following subsections, the human inspiration behind the hierarchical architecture is first discussed (Section 3.1). The structure of the task manager prompt is then formalized (Section 3.2), including the task planning context and dynamic context that capture robot capabilities, constraints, and execution state. The CoMuRoS algorithm is subsequently presented (Section 3.3), which translates human intent into executable robot actions through hierarchical task allocation and execution. This is followed by a description of the robot-level and team-level execution mechanisms (Section 3.4), the human interface for interaction (Section 3.5), and the event detection and classification framework (Section 3.6) that enables event-driven replanning within the proposed architecture. Finally, the curated benchmark dataset and evaluation methodology are presented (Section 3.7) to evaluate generalization across diverse scenarios and heterogeneous robot teams.

### 3.1 Human inspired design

The proposed CoMuRoS architecture draws inspiration from hierarchical structures commonly observed in human organizations. In such organizations, a Chairman or CEO provides high-level instructions in natural language to a manager, who leverages reasoning capabilities along with knowledge of the capabilities of subordinates to allocate tasks, form teams, and devise execution plans. Research on shared mental models in human teams has shown that effective coordination emerges from a shared understanding of team members’ roles and capabilities rather than constant explicit negotiation (Mathieu et al., 2000). Specifically, Mathieu et al. (2000) identified two types of shared mental models critical for team performance: a *task model* (shared understanding of goals and how to achieve them) and a *team model* (shared understanding of who has what capabilities). The subordinates, upon receiving instructions, independently reason to generate low-level executable plans based on their individual skill-set. This results in a hierarchical multi-agent system where reasoning occurs at two distinct levels: high-level task allocation at the manager level and low-level task execution at the subordinate level.

CoMuRoS adopts a similar hierarchical paradigm. The task manager acts as the centralized “manager” agent, maintaining both a task model (encoded in the task planning context) and a team model (encoded in the scenario configuration with each robot’s capabilities, morphology, and constraints), directly realizing the shared mental model structure shown by Mathieu et al. (2000) to improve team coordination and performance. The robots, analogous to subordinate employees, perform decentralized reasoning to generate executable plans tailored to their skills. In this framework, Large Language Models (LLMs) enable reasoning and decision-making at both levels, supporting both task allocation and execution. This hierarchical design naturally facilitates centralized planning combined with decentralized execution.

Human organizations are inherently resilient to disruptions and capable of adapting to events that affect ongoing plans. Salas et al. (2005) identify backup behavior, where team members assist one another when a teammate encounters difficulty, and adaptability as two of the five core components of effective teamwork. A key mechanism enabling this resilience is the proactive reporting of relevant events by subordinates to the manager, allowing the manager to revise plans and reassign tasks to ensure overall goal completion. CoMuRoS incorporates this principle by enabling robots or external cameras to continuously monitor the system, where task allocations are provided as contextual input. Using this context along with sensor inputs and reasoning models (LLMs or VLMs), they determine whether observed events are relevant to the ongoing tasks. When a relevant event is identified, it is proactively reported to the task manager, which triggers a replanning process and generates updated task allocations, realizing the backup behavior and adaptability identified by Salas et al. (2005) in an autonomous multi-robot setting.

**FIGURE 1:**
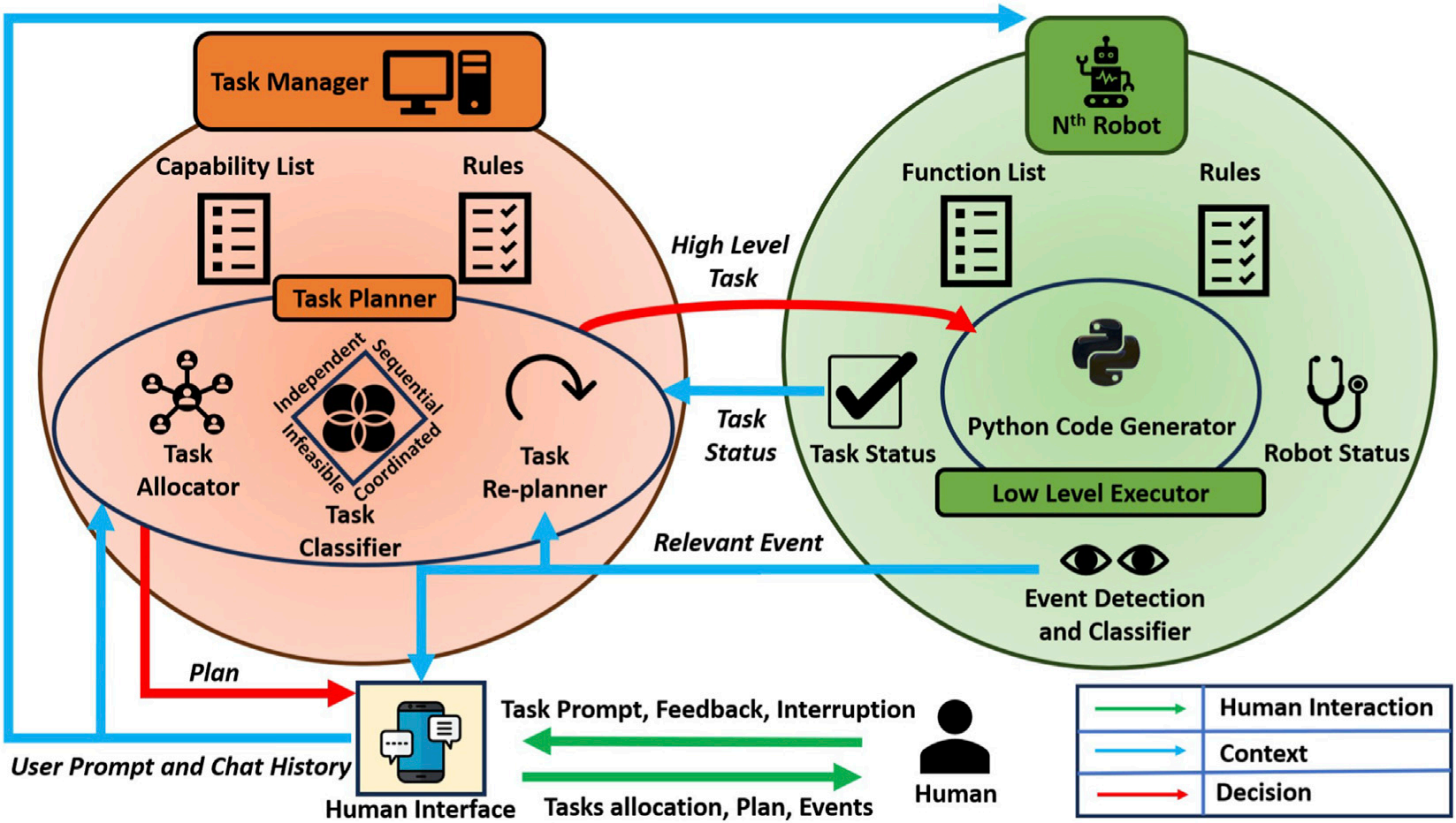
Hierarchical architecture of CoMuRoS.

Figure 1 illustrates this human-inspired design. The architecture follows a hierarchical structure comprising a centralized, event-aware task manager and decentralized robot execution nodes that interact continuously during task execution. As shown on the left side of the figure (orange block), the task manager interfaces with the human, receiving high-level instructions along with contextual information such as chat history. It encapsulates core functionalities including task planning, task allocation, task classification, and task re-planning. These components operate over predefined capability lists and planning rules, enabling the system to interpret natural language instructions, classify tasks into execution types (independent, sequential, coordinated, or infeasible), allocate them to appropriate robots, and adapt plans dynamically in response to evolving conditions.

On the right side of the figure (green block), each robot is equipped with a decentralized execution node consisting of a python code generator LLM and a low-level executor. This module translates high-level task directives into executable programs tailored to the robot’s capabilities. Each robot maintains its own function list, execution rules, and internal state (robot status), ensuring modularity and autonomy. Additionally, an *event detection and classification module* continuously monitors the environment to identify events that may impact task progression.

Consistent with the interaction between managers and employees in human organizations, communication between the task manager and robots is bidirectional. High-level task specifications generated by the task manager LLM are transmitted to the robots, while robots provide feedback in the form of task status updates and detected events. Upon receiving a relevant event, the task manager updates the contextual information and triggers a replanning process to revise task allocation and execution strategies accordingly.

### 3.2 Task manager prompt

**FIGURE 2:**
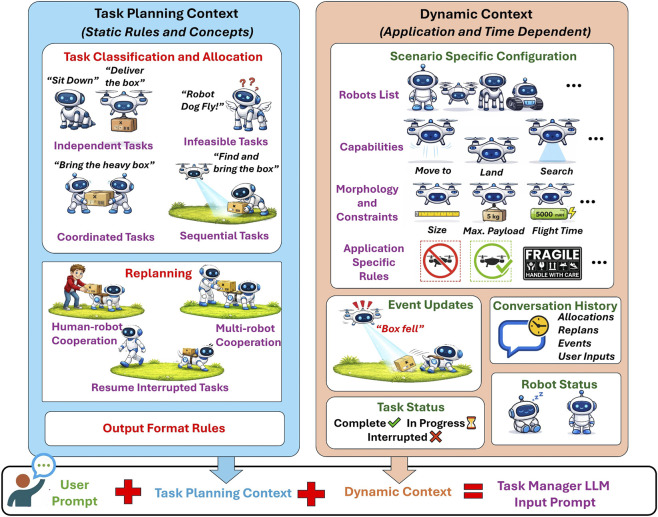
Task manager input prompt is constructed using task planning context, dynamic prompt and user prompt.

The design of task manager prompt is conceptually inspired by the separation between long-term knowledge and working memory in human cognition, where stable knowledge is reused across tasks while context-specific information is dynamically updated. While not a direct biological model, this abstraction provides a useful framework for achieving generalization and adaptability in multi-robot systems. In CoMuRoS, the input prompt to the task manager LLM is structured into three distinct components: the *user prompt*, the *task planning context*, and the *dynamic context* as shown in the bottom part of Figure 2. The user prompt captures the high-level instruction or intent expressed in natural language. The task planning context (long-term knowledge) encodes invariant reasoning structures, including definitions of task types (independent, sequential, coordinated), task allocation strategies, coordination constraints, replanning rules, and output formatting policies. This task planning context serves as a repository of reusable logic applicable across a wide range of scenarios. The dynamic context, in contrast, contains scenario-specific and time-varying information such as available robots with their capabilities, environment conditions, task and robot statuses, detected events, and interaction history. This structured separation ensures that the core reasoning logic remains consistent and reusable, while updates in the dynamic context inform the system of changing situations, enabling the task manager to adapt its decisions accordingly. Consequently, the same planning intelligence can be reused across diverse applications. For a new scenario or deployment, only a scenario configuration file capturing environment specific settings, available robots, their capabilities, and application level constraints needs to be defined or tuned, without modifying the task planning context.

As a result, the system achieves modularity and scalability, where domain adaptation is handled through the *scenario configuration* within the dynamic context, while the underlying multi robot reasoning framework remains unchanged and reusable across applications. During deployment, no modifications are required to the Task Planning Context; only the scenario configuration file, which forms part of the dynamic context (shown in the peach colored box in Figure 2), is updated. The task planning context (shown in blue) is designed to operate in a zero shot manner across new applications.

We demonstrate the generalizability of this task planning context across diverse scenarios in Section 5, using a dataset of scenario configurations and tasks described in Section 3.7. Due to space constraints, the complete task planning context prompt and the full set of scenario configuration files for hardware, simulation, and evaluation are provided on the project website CoMuRos.github.io. Key components of the prompt are presented in the following sections and are also illustrated conceptually in Figure 2.

#### 3.2.1 Task planning context

The task planning context represents the static, reusable component of the prompt and encodes the core reasoning logic for multi-robot task planning. It is independent of any specific environment or robot instance and acts as a repository of reusable definitions and rules governing task classification, allocation, and replanning as shown in the blue rectangle on left in Figure 2.

At the start of the prompt, the task manager is assigned a role:

“You are a task manager AI for a team of robots. Your job is to ensure that all assigned tasks are completed efficiently by distributing work among the robots based on their capabilities.”

This establishes the objective of capability-aware task allocation and completion. Task planning context has the following major components:

**1. Task Classification:** A key component of the task planning context is task classification, which determines how tasks are structured and should be executed temporally. The system distinguishes between multiple task types:

**1a. Independent Tasks:** In the prompt the independent tasks are defined as:

“This format should be applied for both task allocation during planning and replanning, if a task or tasks involves only one robot or can be performed independently.”

As shown in Figure 2, examples include actions such as *“Sit Down”* and *“Deliver the box”*, where tasks can be assigned to individual robots without requiring coordination or temporal dependency.

**1b. Sequential Tasks:** Inside the task planning context prompt sequential tasks are defined as:

“If the task requires multiple robots to COORDINATE to do Tasks that cannot be done parallel and have sequential dependency, ONLY THEN generate a step-by-step plan in this format Else Independent tasks.”

This is exemplified in Figure 2 where a drone first performs *“Find the box”* followed by another robot executing *“bring the box”*, demonstrating temporal dependency between tasks.

**1c. Coordinated Tasks:** In the prompt coordinated task are defined as:

“If task involved requires tight COORDINATION of movements done parallel by robot then mention it in this format. The motion of robots is tightly coupled with each other.”

In Figure 2, this is illustrated through the example *“Bring the heavy box”*, where two robots must act together simultaneously, requiring synchronized execution and joint task allocation. Such joint task allocation is managed by a *team manager* Node, as discussed in Subsection 3.3.

Note that every task is classified as either a sequential task or an independent task. A coordinated task can appear within either category. For instance, a coordinated task may be part of an independent allocation, such as *“Sit down Quadruped”* and *“Other robots together lift the heavy box”*, where the coordinated action is independent of other tasks. Alternatively, a coordinated task can be a subtask within a sequential plan. For example, *“Find the heavy box and bring it to me”* is classified as a sequential task, where the drone first locates the box, followed by a coordinated action in which two robots jointly lift and transport it.

**1d. Infeasible Tasks:** In the task planning context, infeasible tasks are defined in the task planning context prompt as:

“If a robot is given a task that exceeds its payload, reach, or other constraints such as components, print why it cannot perform the task before stating it is unfeasible. Ensure this reasoning is clear before providing alternatives or rejecting the task. However, if an alternative robot can perform the task instead, assign it and print a note.”

In Figure 2, this is illustrated by the example *“Robot Dog Fly!“*, where the task manager must reject the task based on robot morphology and constraints.

**2. Replanning:** The task planning context encodes replanning strategies that are invoked when the current plan must be modified due to an event or user feedback. The prompt specifies that:

“Re-planning should be such that all robots tasks are complete eventually including the tasks that were in progress before the event.”

It further enforces cooperative recovery behavior in response to relevant events through the following rules:

*“When an event is RELEVANT, you MUST begin the Plan by issuing ‘STOP’ to ALL robots involved, including those with IN PROGRESS or COMPLETED tasks.”* and *“In case of failure let robots HELP each other based on their capabilities.”*

Figure 2 illustrates this in the replanning section through an example scenario where a drone detects a box falling off the quadruped and reports the event. This triggers a transition to multi robot cooperative recovery or human assisted execution. Multi robot cooperative recovery refers to robots assisting each other based on their capabilities to complete the task, whereas human assisted execution involves requesting intervention from a human when the task cannot be completed autonomously.

These behaviors are demonstrated in the hardware experiments in Section 4.1.1 and Section 4.1.2, respectively. Human-robot collaboration is explicitly supported through:

“When the task is difficult to continue because of Robot Morphology or Robot Capabilities then you can ask for assistance from the human or user.”

Additionally, the task planning context enforces execution continuity before and after disruptive events through resume rules:

“Resume must be strictly based on the current status, not task history. If tasks are interrupted and clearly marked as ‘IN PROGRESS’ at the time of the event, they should be resumed. Ignore old tasks if they are marked as ‘TASKS COMPLETE’ in the current robot state.”

As illustrated in Figure 2 under the replanning section, after assisting the quadruped, the humanoid and quadruped resume their original tasks.

**3. Output Format:** Finally, the prompt defines structured output requirements through explicit formats which can be processed by a deterministic algorithm to parse type of robot task and robots involved in allocations and their corresponding tasks for further execution and communication. The format mentions *Plan* for sequential tasks and *Independent Tasks* for independent tasks. For coordinated tasks the task is assigned to a team and team composition is mentioned. An example output format for task which is classified as independent and coordinated is shown below:

Independent Tasks:
Robot_Name_1: Stand and sit 10 times,
dance,...
Robot_Name_2: Go to charging station, task_2_name,...
Team 1 [Robot_Name_6, Robot_Name_7, Robot_Name_10]:
Three robots go to a chair together,...
Robot_Name_3: Pick and place trash, move forward 5 m,...
Team 2 [Robot_Name_8, Robot_Name_12, Robot_Name_15]:
Three robots go to a chair together, .
multirobot_list:
[]
singlerobot_list:
[Robot_Name_1, Robot_Name_2, Team 1, Robot_Name_3, Team2]

The single robot list indicates, that task allocations of robots and teams will be dispatched parallely to the robots or team manager respectively. Similarly multi-robot list contains names of teams or robots involved in sequential task and subtasks are dispatched to robots or team as per the queue decided by task manager.

Overall, the task planning context encapsulates reusable reasoning primitives that govern task interpretation, decomposition, allocation, and adaptation during execution.

#### 3.2.2 Dynamic context

The dynamic context represents the application dependent and time varying component of the prompt and functions as the working memory of the task manager. It provides the situational information required to instantiate the abstract planning rules defined in the task planning context.

**1. Scenario Configuration:** As shown in Figure 2, the dynamic context contains scenario specific configuration, which captures environment and deployment dependent parameters. This includes the list of available robots, their capabilities, morphology based constraints and application specific rules.

**1a. Robot List and Capabilities:** The list of robots contains the names and types of robots, for example, *“Quadruped: RoboDog, Drone: Drone1, Humanoid: Humanoid1, Humanoid: Humanoid2, … “*. Note that this list is part of the dynamic context; hence if during deployment a new robot becomes available or an existing robot is disabled, this list can be updated. The same applies to other information in the scenario configuration. For each robot, its capabilities are described in natural language. For example,: *“Quadruped: RoboDog: Can walk, sit, jump but cannot back flip or handstand, please do not assume it.”* If any robot has coordination capabilities such as bimanual manipulation of objects or formation control, these should also be mentioned in the capabilities list. *“Humanoid: Humanoid1: Walk, Sit, Stand, Pick, Place, Collaboratively pick objects with other humanoids of same kind, … etc.,”*

**1b. Application Specific Rules:** Application specific rules enable users to customize the use of CoMuRoS for their application by providing additional information and constraints imposed by the environment and tasks. For example, *“Region A is a no fly zone”, “Boxes carry fragile components handle with care.“, “Boxes labeled heavy have weight of* 30 kg*” and “Co-ordinates for delivery are 4,0 unless mentioned otherwise” …* These, combined with robots’ morphological and operational constraints and capabilities, ground the abstract task planning context to the reality of the application or scenario.

**1c. Morphology and constraints:** The most important aspect of the configuration file is to mention the morphology and operational constraints of each robot. This enables the task manager to infer infeasibility of a task and plan tasks around it. For example, *“Drone: Drone 1: Payload:* 5 kg*, Flight time: 30 min”, Size: …. Humanoid: Payload Capacity:* 20 kg*, Height: 1.5 m, Reach: …etc.,”*. This enables execution or rejection of tasks such as *“Robotic arm please keep the book on the robot dog”*, depending on the reach of the robot and the height of the robot dog. It can also reject tasks such as “drone deliver the heavy box”. Note that the definition of a heavy box and delivery location should be mentioned in the application specific rules. This also enables the triggering of coordinated tasks if the task violates constraints of individual robots but coordination skills are available, as specified in the capabilities section of the scenario configuration. For example, “Deliver the heavy box” will lead to task allocation as “Team1 [Humanoid1, Humanoid2]: Deliver the heavy box”, as the task manager LLM can reason based on the context in the configuration file that together humanoid 1 and humanoid 2 have a payload capacity of 40 kg and can lift a box of 30 kg. Scenario configuration also has the initial states of the robots.

Apart from the scenario configuration, evolving situational information including *robot status*, *task status*, *conversation history*, and *events* are incorporated into the dynamic prompt. This information allows the task manager node to perform context-aware task allocation and distribution by considering the current execution state of the system.

**2. Robot Status:** Apart from the scenario configuration file, task status and robot status can include additional information about the current robot state, which can be useful in execution of certain tasks. For example, if objects can be loaded onto a quadruped only in a sitting state, it is useful for the robot state to contain information about whether the robot is standing or sitting. Robot status can also contain information about battery status, current availability of the robot, etc., which can be useful for task allocation by the task manager or for execution of tasks by robots. Initial robot status is mentioned in the scenario configuration file. During execution robot status should be updated by the robot executable skills during execution or some external sensing agent like CCTV. In this paper each skill is associated with a post-condition that results in update of robot status. For example, the skill sit() updates robot status from *“standing”* to *“sitting”*.

**3. Task Status:** Every task, irrespective of its type, is associated with a status variable that can take one of the following values: COMPLETED, INTERRUPTED, or IN PROGRESS. Each skill or executable function (e.g., walk, run) in the robot skill set is designed to return the task status based on feedback derived from the robot’s sensing capabilities. In scenarios where sufficient sensing is unavailable, the task status may be inferred in an open-loop manner. Additionally, if relevant sensing information is available externally (outside the robot), it can also be utilized to update the task status.

Upon receiving a relevant event alert, the task manager stops all robots whose task status is IN PROGRESS and updates their respective task statuses to INTERRUPTED. During subsequent replanning, only those interrupted tasks that remain relevant after the event are considered for resumption, typically after completing any newly introduced subtasks resulting from the replanning process. Tasks with COMPLETED status are excluded from replanning. In case of sequential tasks, next task in plan is allocated to the corresponding robot only after current task status shows COMPLETED.

**5. Conversation history:** Conversation history contains rich context of previous events and user interactions, enabling temporal context. Tasks such as *“Repeat the previous task”* or *“Bring it to me”* can be executed due to temporal context, enabling user interaction to remain natural, as a user would not like to repeat instructions or over specifying a task (Note that *“it”* in *“Bring it to me”* can be inferred from the conversation history). Conversation history is also useful in event detection, allowing robots to understand what affects current tasks and what can be ignored. For example, a user command *“I do not want it anymore”* should trigger replanning, preventing the robot from delivering the object needed earlier based on previous conversation history.

These components together provide the situational awareness required for adaptive decision making. When events occur, the dynamic context is updated and the task manager generates revised plans using the same task planning rules without modifying the underlying logic. Event detection and classification will be discussed in upcoming Section 3.6.

A key design feature of CoMuRoS is that adaptation to new scenarios requires modification only to the dynamic context, specifically the scenario configuration. The task planning context remains unchanged, continuing to provide definitions such as *“Independent Tasks”*, *“Coordinated Tasks”*, *“*Re *planning Rules”* etc. This enables reuse of the same planning intelligence across diverse applications.

Overall, the separation between the task planning context and the dynamic context allows CoMuRoS to maintain a consistent reasoning framework while adapting to new environments and task requirements, achieving modularity, scalability, and generalization in multi robot systems.

### 3.3 CoMuRoS algorithm

**FIGURE 3:**
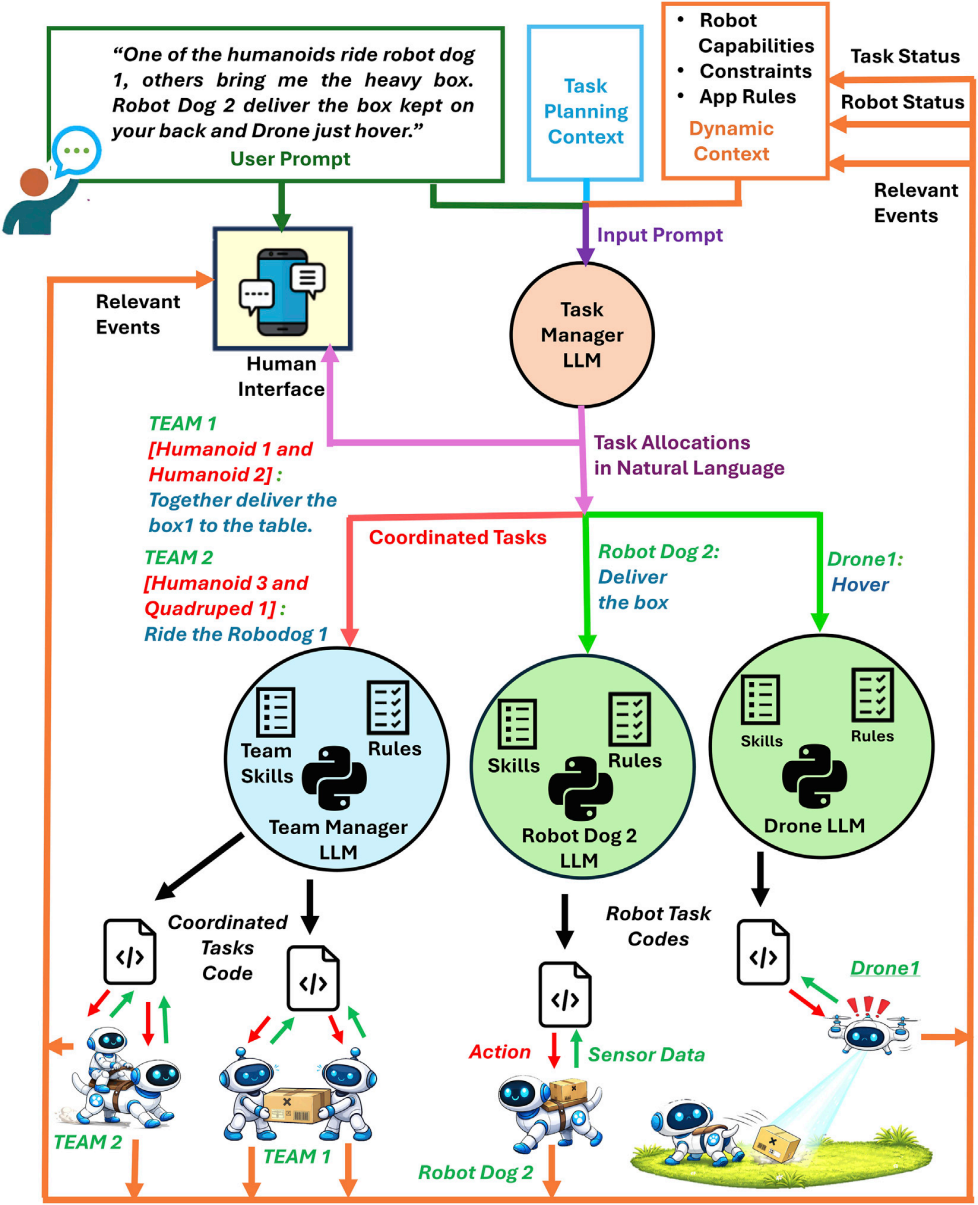
Flow of information through the components of CoMuRoS architecture human interface, task manager, team manager, robots, dynamic context and task planning context.

Human intent, expressed through the *human interface* (Figure 3), is first translated into high level plans that remain in natural language.

As shown by the green arrows originating from the *user prompt* in Figure 3, the user instruction is forwarded to the task manager LLM, where it is fused with structured contextual inputs. These inputs include the Task Planning Context (depicted with a blue block) and the Dynamic Context (depicted with an orange block), both of which are combined through the purple “Input Prompt” flow into the task manager. The Task Planning Context provides general knowledge such as task definitions, allocation strategies, and coordination principles, while the Dynamic Context continuously aggregates robot capabilities, constraints, application rules, task and robot status, and relevant events (highlighted by orange feedback arrows).

Based on this combined input, the task manager LLM generates high level task allocations in natural language, which are propagated downward through purple arrows indicating semantic task assignments. These allocations distinguish between independent robot tasks and coordinated multi robot tasks. For individual robots, such as Robot Dog 2 and Drone 1 the task allocation is shown using green arrows and for team allocation in case of co-ordinated tasks red arrow is used as shown in Figure 3,. These high level natural language task allocations (e.g., “Deliver the box” or “Hover”), are then passed to the respective robot specific LLMs.

Each robot LLM integrates the allocated task with its internal knowledge context of skills and rules (illustrated within each green circular module in Figure 3). This process results in executable task code, shown via document icons, whose execution produces low level control actions. The execution loop is explicitly depicted using red and green bidirectional arrows, where red arrows represent actions (e.g., velocity commands) sent to the robot, and green arrows represent incoming sensor data (e.g., odometry or perception feedback). This closed loop interaction ensures that execution remains responsive to real time environmental conditions.

For coordinated tasks, indicated by the red arrow labeled “Coordinated Tasks” in Figure 3, the task manager additionally specifies team composition along with the task description. This information is routed to the team manager LLM, which operates on the context of team level skills and coordination rules (shown in the circular blue module on the left). For each task, the team manager synthesizes centralized coordinated task code that enforces synchronization among team members.

A continuous feedback mechanism is maintained throughout the architecture. Execution status, task progress, and environmental observations are propagated back into the Dynamic Context through orange feedback pathways, ensuring that the system maintains an up to date representation of the world. Additionally, relevant events, either observed directly or inferred through robot level event detection modules (not explicitly shown in Figure 3), are also injected into this Dynamic Context. Notably, robots also have access to conversation history (not shown in Figure 3 due to space constraints and discussed in a subsequent section), which provides temporal context for interpreting ongoing interactions; leveraging this history, robots can proactively classify and report relevant events rather than reacting purely to instantaneous observations. These updates enable the task manager to perform event driven replanning when necessary, closing the loop between perception, reasoning, and action.

Overall, the color coded flows in Figure 3 convey the architecture’s information dynamics: green for user input and sensor feedback, purple for high level semantic reasoning and task allocation, red for coordinated task pathways and low level actions, blue for static planning knowledge, and orange for dynamic system state and feedback. This structured flow enables CoMuRoS to seamlessly bridge high level human intent with context aware, and adaptive multi robot execution.

The running example in Figure 3 demonstrates how a complex natural language instruction is processed and executed through the CoMuRoS architecture. The user provides the command *“One of the humanoids ride robot dog 1, others bring me the heavy box. Robot Dog 2 deliver the box kept on your back and Drone just hover.”* via the human interface, which is passed to the task manager LLM as shown using green arrows. The task manager combines this input with the Task Planning Context and Dynamic Context to interpret the instruction and generate structured task allocations, which are indicated using purple arrows.

The instruction is decomposed into coordinated and independent tasks. Two teams are formed for tasks that require synchronized motion: Team 1 (Humanoid 1 and Humanoid 2) is assigned to jointly deliver a box to a table, and Team 2 (Humanoid 3 and Quadruped 1) is assigned the task of riding the robot dog. These coordinated tasks are sent to the team manager LLM, as indicated using red arrows, where synchronized execution code is generated based on team level skills and rules as context for the team manager LLM.

In parallel, tasks are assigned to individual robots. Robot Dog 2 is assigned to deliver a box, and Drone 1 is assigned to hover. These assignments are processed by their respective robot level LLMs, where task descriptions are combined with robot specific skills and rules to generate executable task code. The execution loop involves red arrows indicating actions (e.g., motion commands) and green arrows indicating sensor feedback, enabling closed loop control.

During execution, robots continuously update task progress, robot status, and observations in the Dynamic Context, as shown using orange arrows. For example, the drone can monitor the environment and report relevant events, which are incorporated into the Dynamic Context. This enables the task manager to perform event driven replanning if required. The example thus illustrates how a single high level instruction is decomposed into coordinated and independent tasks, executed across multiple robots, and continuously monitored through feedback.

The necessity of synchronization in coordinated tasks highlights a key design principle for the architecture: task structure determines the level of centralization required for execution. Tasks involving shared loads, physical coupling, or mutual constraints are elevated to team level reasoning, while individual robot task execution remains decentralized. Throughout execution, all robots report their status and observations to the Dynamic Context indicated by orange arrows in Figure 3. For example, the hovering drone may observe deviations or unexpected events and proactively report them, enabling the task manager to re evaluate task allocations. Figure 3 demonstrates that at the top level of the hierarchy, the centralized task manager develops high level plans, while at the bottom, robots and team managers reason about task execution based on individual skills.

This discussion shows the topological flow of information through the architecture; there is also a temporal aspect to the allocation of high level tasks based on task classification. The temporal aspect of the hierarchical architecture as described in Figure 3 is explained in Algorithm 1.

Algorithm 1 Task Manager Classification, Allocation, and Event-Driven Replanning.

**Require:** User prompt P , scenario config C , chat history H , task status S , Event E

**Ensure:** Task plan Ψ mapping each robot to its assigned subtask(s)
  
// *Prompt Construction*
 1: $\rho_{s}\leftarrow$ *StaticPrompt*: rules and definitions for cooperative planning
 2: $\rho_{d}\leftarrow$ *DynamicPrompt*: {*C.* robots, *C.* capabilities, *C.* morphologies, *C.* rules, *H*, *E*, *S*}
  
// *Classification + Allocation: single LLM call*
 3: $\Psi \leftarrow LLM\left(\rho_{s},\;\rho_{d},\;P\right)$

  
// *task type and assignments produced jointly;* Ψ *explains if infeasible*
  
// *Execution*
 4: **if** Ψ.type=INDEPENDENT **then**
 5:  **for** each subtask τ in Ψ **do**
 6:   **if** τ is coordinated **then**
 7:    Dispatch τ to Team Manager
 8:   **else**
 9:    Dispatch τ to assigned Robot’s LLM
 10:   **end** **if**
 11: **end** **for**
 12: **else** **if** Ψ.type=SEQUENTIAL **then**
 13:  **for** each step k in Ψ.plan **do**
 14:   **if** Ψ[k] is coordinated **then**
 15:    Dispatch Ψ[k] to Team Manager
 16:   **else**
 17:    Dispatch Ψ[k] to assigned robot’s LLM
 18:   **end** **if**
 19:   **await** robot status ∈{COMPLETED, INTERRUPTED}

 20:  **end** **for**
 21: **end** **if**
  
// *Event-Driven Replanning: triggered on relevant event* E

 22: **if** event E received on chat log **then**
 23: H←APPEND(H, E)

 24: S←UPDATESTATUS(Ψ)

  
// *robots report completed/inprogress/interrupted*
 25: $\rho_{d}\leftarrow$ {*C.* robots, *C.* capabilities, *C.* morphologies, *C.* rules, *H*, *E*, *S*}
 26: $\Psi^{\prime }\leftarrow LLM\left(\rho_{s},\;\rho_{d},\;E\right)$

   
// *LLM uses* S *and* H *to re-classify, re-allocate, issue stop/resume,*
   
// *skip completed robots, assign to human if beyond robot capabilities*
 27:  **await** human completion event (if any); update H ; continue $\Psi^{\prime }$

 28: $\Psi \leftarrow \Psi^{\prime }$ ; **goto** line 6
 29: **end** **if**
 30: **return** Ψ

The task manager operates on the inputs (P,C,H,S,E) , where P denotes the user prompt, C encodes the scenario configuration (robots, capabilities, morphologies, and rules), H is the accumulated interaction history, S represents the current task status, and E denotes a detected event. The prompt provided to the LLM is constructed by combining a static component $\rho_{s}$ , which encodes task planning rules and definitions, with a dynamic component $\rho_{d}=\left(C.\text{robots},C.\text{capabilities},C.\text{morphologies},C.\text{rules},H,E,S\right)$ that captures the current system context. The task manager then invokes the LLM as $\Psi \leftarrow L_{M}\left(\rho_{s},\rho_{d},P\right)$ , which jointly performs task classification and allocation, producing a structured plan Ψ that maps each robot μ to its assigned subtask(s) and specifies the execution type.

The execution strategy is determined by Ψ.type . For independent tasks, subtasks are dispatched concurrently. For sequential tasks, subtasks are issued in order, where each step Ψ[k] is processed sequentially and the system waits for updated execution feedback through S←UPDATESTATUS(Ψ) before proceeding. Each subtask is dispatched either to a robot-level LLM or to the team manager depending on whether the subtask is coordinated.

Upon receiving a relevant event E , the task manager updates its internal context as H←APPEND(H,E) and S←UPDATESTATUS(Ψ) , thereby incorporating both interaction history and the latest execution state. A revised dynamic prompt is then constructed using the updated (C,H,E,S) , and the LLM is invoked again to generate an updated plan $\Psi^{\prime }\leftarrow L_{M}\left(\rho_{s},\rho_{d},E\right)$ . This replanning step enables the system to reclassify tasks, reallocate subtasks, and adjust execution flow in response to changing conditions, while implicitly preserving progress by conditioning on S and H . The updated plan replaces the previous plan, and execution continues accordingly.

### 3.4 Robot and team manager level execution

The architecture supports heterogeneous platforms. Although the focus of this paper is on multi robot applications, a robot can be replaced by any agent that can react to signals provided by the task manager and, if required, has reasoning abilities using LLMs or other methods to perform tasks. Non robot agents can include airline booking systems, smart home devices, or human employees receiving orders from the task manager LLM. In the context of multi robot systems, each robot receives high level tasks in natural language from the task manager.

As shown in Figure 1, each robot node maintains a dictionary of skills or execution functions with associated descriptions (inputs, outputs, and use cases). For example, for a mobile robot,

*Skill 1: goto-position, Function:* *goto(position)* *, Description: The robot moves to a specific position in 2D using PID control on odometry values. Example usage:* *goto([5,4])* *.*

*Skill 2: stop, Function:* *stop()* *, Description: Stops the robot motion immediately.*

These functions may correspond to reinforcement learning policies, imitation learning models, Python code, or ROS nodes. Implementation-specific skill libraries for the hardware and simulation experiments are provided in Supplementary Appendix A. These libraries use ROS2- and simulator-based tools including MoveIt, ChoiRbot, Grounding DINO, Unitree Go2 ROS2/SDK interfaces, CHAMP-based locomotion control, IFRA LinkAttacher, and the SJTU drone simulator (Coleman et al., 2014; Testa et al., 2021; Liu et al., 2023; Unitreerobotics, 2024; Jain, 2024; Lee, 2013; IFRA-Cranfield, 2024; Nova, 2025). The robot’s LLM decomposes the high level command and combines relevant functions to generate Python code for execution, thereby producing a ready to run low level plan. This approach is conceptually aligned with “Code as Policies” (Liang et al., 2023), where language models generate executable code to compose robot skills from modular primitives. The complete skill or function libraries for all hardware and simulation experiments are provided in Supplementary Appendix. Tasks that are classified as independent or sequential and involve one robot are handled by the robot’s LLM. Coordinated tasks allocated to a team of robots are routed to the team manager LLM.

**Team Manager:** Just as a robot node has access to executable functions of robot skills, the team manager node has access to a dictionary of coordination skill functions and their descriptions. For example,:

*Skill 1: Formation Control. Function:* *goto-pos(robotnames, start, goal, shape)* *. Description: Allows mobile robots to move in formation to a goal. Robots first form the specified shape and then move collectively. Only {robot1, robot2, robot3} are compatible with formation control.*

*Skill 2: Bimanual Pick of Object. Function:* *bimanual-pick(robotnames, object_id, grasp_points)* *. Description: Enables two compatible robotic arms to collaboratively pick and transport an object that cannot be handled by a single robot. The robots coordinate grasp points and synchronize their motion to ensure stable lifting and transport. Inputs include robot names, object identifier, and grasp points. Only robots with compatible manipulators can execute this skill. Example usage:* *bimanual-pick([robot4, robot5],* *box1* *, [left_handle, right_handle])* *.*

As shown in Figure 3, the team manager reasons based on the context of team level skills and coordination rules, combined with team composition and tasks. For each team, the team manager synthesizes centralized coordinated task code that enforces synchronization among team members. The Python code for coordinated tasks is executed on a central server, and only low level velocity commands are provided to robots.

Robots are agnostic to whether tasks are sequential or independent. Each robot processes one high level command at a time and, upon completion, updates the task status to COMPLETED. Execution functions must include mechanisms to update both task and robot status, either through sensor feedback or open loop logic. Robot status may include descriptors such as “sitting,” “standing,” or coordinates such as (4, 0).

Robots are also responsible for proactively detecting and reporting events in their environment using onboard sensors or external inputs (e.g., CCTV). Event detection operates in parallel with task execution. An LLM is employed to classify detected events as relevant (affecting task progress) or irrelevant (e.g., “a dog is barking” when robots are tasked with indoor cleaning). Relevant events are reported to the task manager, which triggers replanning. Event detection methods may include image processing, action recognition, or vision language models.

### 3.5 Human interface

**FIGURE 4:**
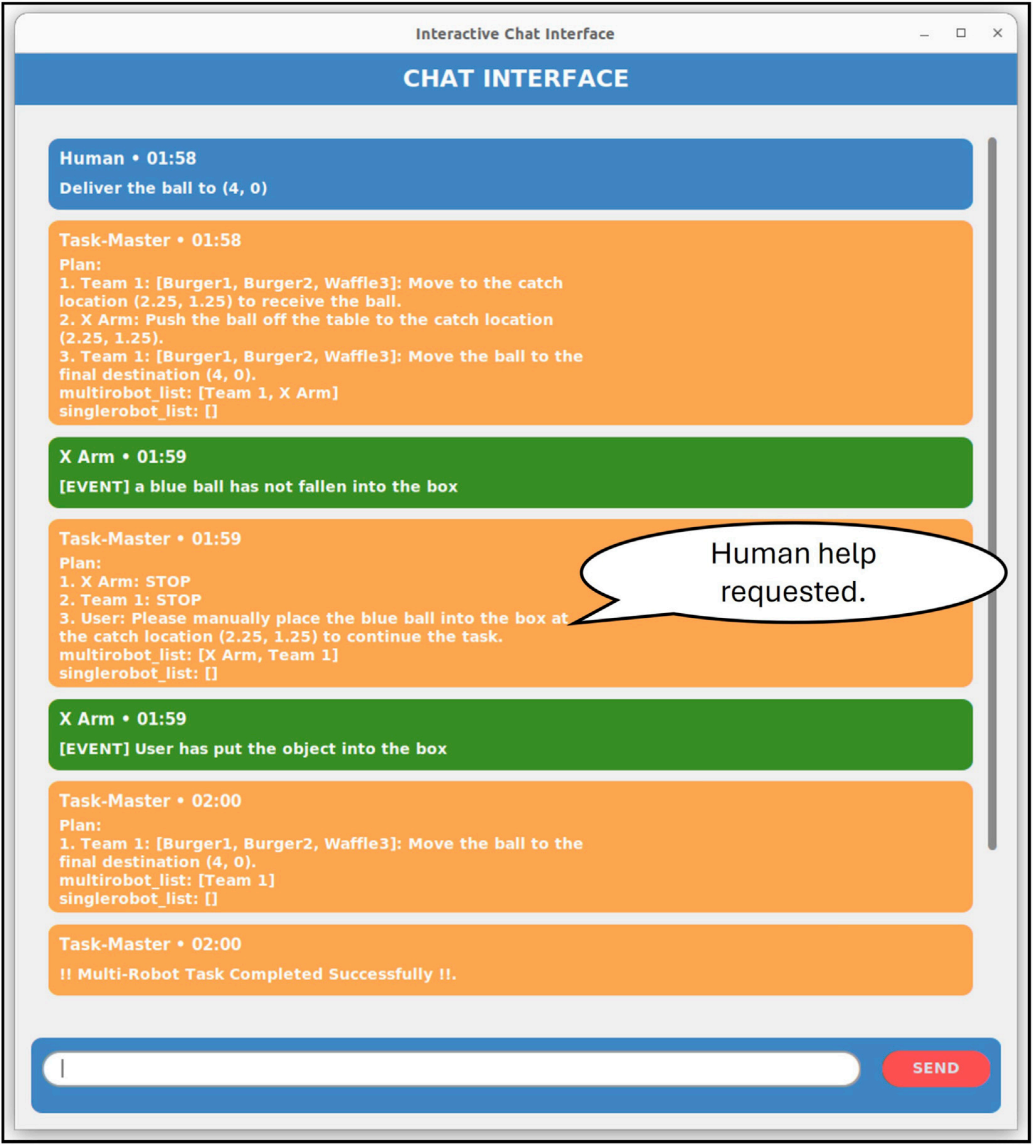
Chat Interface for human robot collaboration. User can communicate with multirobot system using textbox at bottom of interface. Events are shown in green strips. Communication by user is shown in blue strip and orange strip contains task manager response. This example contains task allocations, replans, human inputs, events and task allocation to human for a hardware experiment where human helps a formation carrying a box described in subsection 4.1.2.

A chat-based interface enables continuous natural language communication between humans and the multi robot system, as shown in Figure 4. Through this interface, the user can provide information, give new commands, interrupt ongoing tasks, or change intention at any time during execution. The task manager can also ask the user to assist robots or, if the human is part of the team, allocate tasks to humans through this interface. The dialogue is color coded, with user commands in blue, task allocations from the task manager in orange, and robot detected events in green, all displayed in temporal order. The chat history is preserved and provided as context in the dynamic prompt, as discussed earlier, so that instructions like “repeat the earlier task” or “pick it up again” can be interpreted correctly. User input is given by typing in the interface, but it can be extended to speech, and multilingual interaction is also supported depending on the LLM used.

### 3.6 Event classification

**FIGURE 5:**
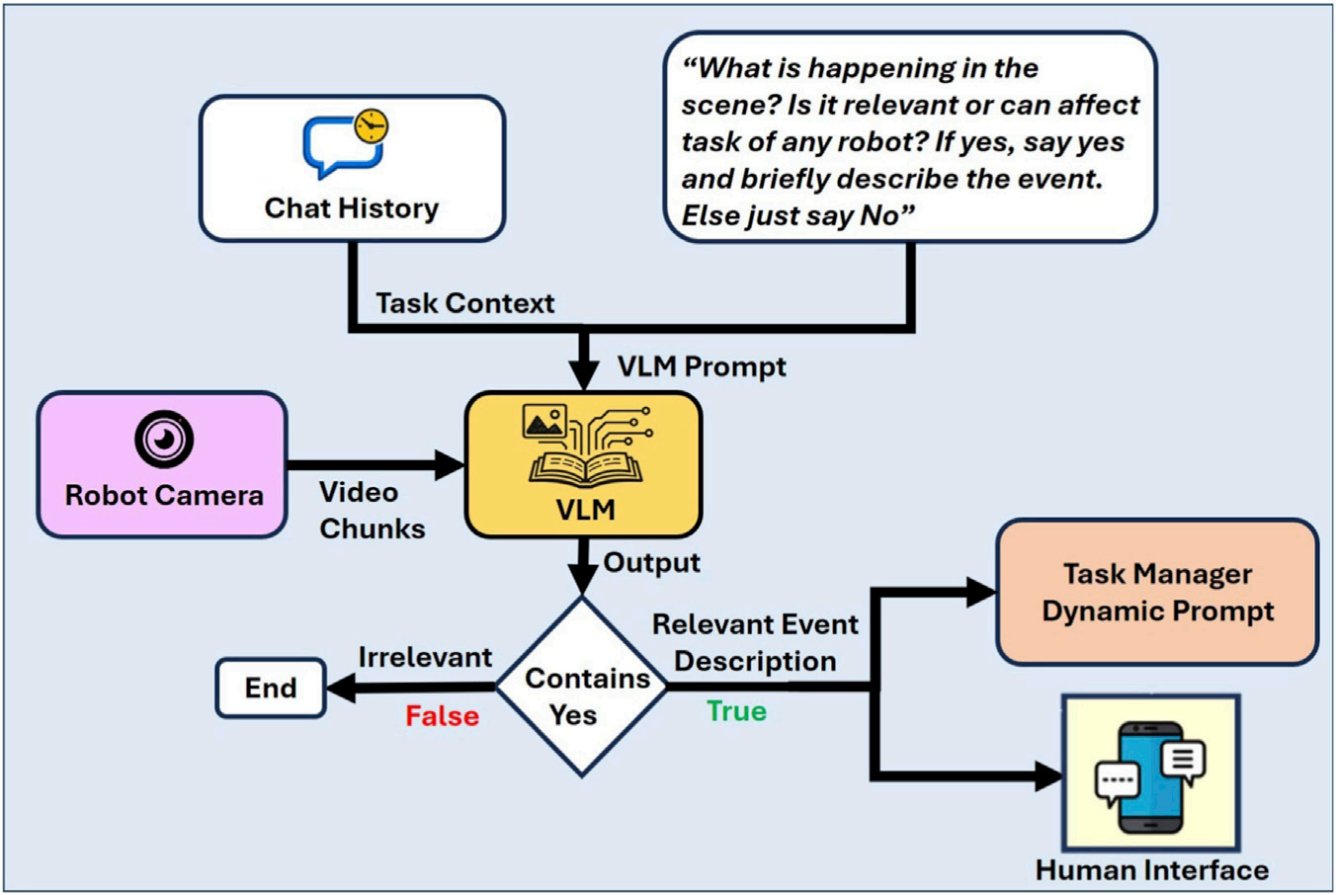
Figure shows a naive algorithm of how events are classified as relevant and reported proactively to the task using VLMs.

In addition to task planning and execution, CoMuRoS incorporates an event-detection mechanism that continuously runs to identify changes in the environment that may influence ongoing tasks. Figure 5 illustrates one possible realization of this mechanism using a VLM used in this paper. In this setup, the robot’s onboard camera streams visual observations in the form of video chunks of predefined size at fixed time intervals, which are processed by a VLM along with a task-aware prompt. The prompt is constructed by combining recent chat history and task context, enabling the model to interpret the scene with awareness of ongoing objectives.

The VLM is queried with a structured instruction: *“What is happening in the scene? Is it relevant or can it affect the task of any robot? If yes, say yes and briefly describe the event. Else just say No.”* This formulation reduces event detection to a binary relevance classification coupled with a mandatory natural language description when an event is deemed relevant. The decision block evaluates the response: if the output indicates “Yes,” the accompanying event description is always propagated to the task manager as part of the dynamic context and is also communicated to the human interface; otherwise, the pipeline terminates without further action. Note that relevant events are communicated by the robots even if they are not allocated any tasks proactively as this pipeline runs continuously on all robots.

The requirement of an explicit event description is critical, as it directly serves as replanning context for the task manager. By encoding events in natural language, the system enables flexible and context-aware reasoning during replanning, without requiring a fixed set of predefined event categories.

The event-detection component is not restricted to VLM-based inference and can be replaced or augmented with alternative approaches, including classical computer vision techniques, image-processing = LLM pipelines (used in hardware experiment in Section 4.1.1), or more sophisticated video understanding methods with improved temporal segmentation and intelligent chunking strategies. This modularity allows the architecture to adapt to different sensing modalities of different robots, computational constraints, and application requirements, while maintaining a consistent interface for replanning.

### 3.7 Dataset and evaluation metrics

**FIGURE 6:**
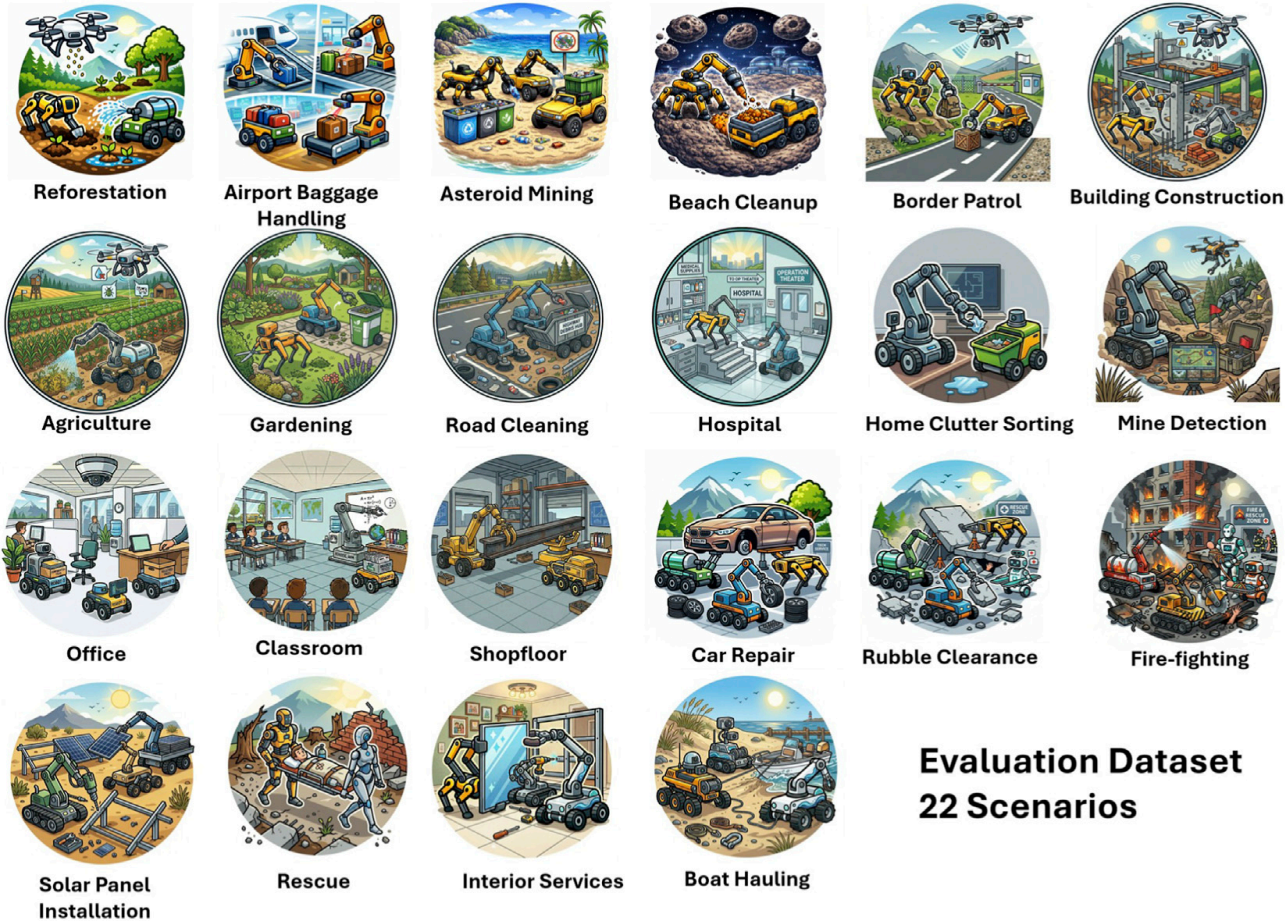
Evaluation dataset is a collection of scenario configurations and tasks across diverse domains.

We constructed a textual dataset of 22 diverse scenarios to evaluate CoMuRoS for generalization across different scenarios and robots. Each scenario is defined by a configuration file. The 22 scenarios have 54 distinct tasks and around 20 robots. The dataset and scenario configuration files contain application specific context for each scenario is available on our website comuros. github.io. Figure 6 illustrates the diversity of scenarios inside the dataset. Scenarios considered for zero-shot evaluation are from different application domains. Task planning context is not altered only scenario configuration files are generated manually with generative AI assistance for creation of the dataset. As we are not altering the core task planning context for any of the scenarios or modify the scenario-configuration files iteratively during evaluation, good performance across these scenario shall confirm zero-shot generalizability of CoMuRoS high level task planning capability for multirobot systems. Performance is defined by the following metrics:

**Task Allocation (TA)** equals 1 if tasks are correctly assigned according to capabilities of all robots and user task, otherwise 0.; **Task Classification (TC)** equals 1 if the classification is feasible given the allocation, otherwise 0 (some tasks may be performed both sequentially and independently); **Intersection-over-Union (IoU)** measures the overlap between the task manager’s plan and a ground truth plan (0-1). If the plan is correct (Correctness = 1), IoU = 1. Otherwise, IoU is computed as the overlap between the generated plan and a ground truth plan selected by the evaluator from the set of possible correct outputs for that task.; **Executability (Exec)** is the percentage of feasible allocations given robot capabilities and constraints (normalized to 0-1); and **Correctness** equals 1 if an expert evaluator judges the plan to succeed, else 0. Correctness is the only metric requiring human judgment; TA, TC, IoU, and Executability are computed deterministically from the scenario configuration and LLM output. Note that the textual benchmark are used to evaluate the task manager LLM’s planning and generalization capability; the hardware experiments in Section 4 constitute the end-to-end system evaluation. The evaluators are student researchers working in the field of robotics. In addition to the 22-scenario task planning benchmark, a separate replanning benchmark of 20 scenarios is constructed to evaluate event-driven replanning capability. Each scenario in the replanning benchmark is defined by an initial user command, a robot team configuration, and an event description that disrupts the initial plan. The task manager first generates an initial plan; upon receiving the event, it generates a revised plan. The revised plan is evaluated using the same metrics (TA, TC, IoU, Exec, Correctness) only when the initial plan is correct and the event is relevant. Each task is evaluated 5 times to account for LLM output variability. As Correctness metric can be prone to human subjectivity inter-rate study is performed between two human-evaluators for this metric on the task planning textual dataset.

The tasks defined for each scenario in the dataset are provided as input to the LLM, together with the corresponding scenario configuration (included in the dynamic context) and the predefined task planning context (static context). The generated outputs for each task–scenario pair are subsequently examined by a human evaluator and assessed using the evaluation metrics described above. To demonstrate the evaluation methodology lets consider Reforestation scenario.

Scenario configuration for reforestation scenario specifies a heterogeneous team.

*“Drone, Robot Dog with Spade, Differential Drive Robot with Sprinkler”* along with detailed capability descriptions. For example, the drone *“can map terrain and locate planting zones; and seed the area using a seed dispenser”*, while the robot dog *“can dig holes in rocky, grassy, or uneven forest terrain”*, and the wheeled robot is *“responsible for watering the newly planted areas”*. Along with capabilities, the file includes morphology (payload, speed, size), initial robot states (e.g., “Idle,” “Full battery”), and environment-specific rules, such as *“All robots should PAUSE the work when animals are found.”* and *“Don’t waste water.“*. It also defines replanning constraints, ensuring that task completion is reassessed dynamically during events.

The tasks defined for each scenario in the dataset are provided as input to the task manager LLM, along with the corresponding scenario configuration (included in the dynamic context) and the predefined task planning context (static context). The generated outputs for each task–scenario pair are subsequently evaluated by a human annotator using the metrics described above. To illustrate the evaluation methodology, we consider the reforestation scenario.

The scenario configuration specifies a heterogeneous team *“Drone, Robot Dog with Spade, Differential Drive Robot with Sprinkler”* along with detailed capability descriptions. For instance, the drone *“can map terrain and locate planting zones and seed the area using a seed dispenser”*, the robot dog *“can dig holes in rocky, grassy, or uneven forest terrain”*, and the wheeled robot is *“responsible for watering the newly planted areas”*. In addition, the configuration encodes morphology (e.g., payload, speed, size), initial robot states (e.g., *“Idle,” “Full battery”*), and environment-specific rules such as *“All robots should PAUSE the work when animals are found”* and *“Don’t waste water.“*.

Consider the user prompt: *“Dig, Seed and Water the seeds in zone A.”* and task manager response,

Plan:
Robot Dog with Spade: Dig holes in zone
A.
Drone: Seed zone A.
Differential Drive Robot with Sprinkler: Water the seeds.

As the task is correctly classified as sequential, TC = 1. The task is allocated according to the capabilities of the robots mentioned in the configuration file, hence TA = 1. The correctness is 1 because the objective of the user can be achieved using this plan, and executability is 1 as the tasks are feasible for all robots. The same task can also be classified as Independent and still achieve the desired result, as shown below:

Plan:
Robot Dog with Spade: Dig holes in zone
A.
Drone: Seed zone A.
Differential Drive Robot with Sprinkler: Water the seeds.

Hence, it is possible that for the same task, TC = 1 for either type of classification. Now consider another possible task manager response,

Independent Tasks:
Robot Dog with Spade: Dig holes in zone
A.
Drone: Seed the holes in zone A.

Even though the *“water the seeds”* task is missing, still TC = 1 (as both Independent and Sequential classifications are possible). TA = 0 as task allocation is incomplete and one of the robots is not assigned a task. Correctness = 0 since the user intention is not fully satisfied, and IoU = 0.66 as there is a 2/3 overlap between the correct response and Output 3. Using the same metrics and methodology tasks in all other 22 scenarios are evaluated and results are presented in Section 5.

## 4 Experiments and results

### 4.1 Hardware

To demonstrate CoMuRoS capabilities in task allocation, event detection, classification, and event-driven replanning with onboard sensors, two sets of hardware experiments were conducted: (1) Multi-robot collaboration with replanning, and (2) Human-formation collaboration.

#### 4.1.1 Multi-robot collaboration using event-driven replanning

**FIGURE 7:**
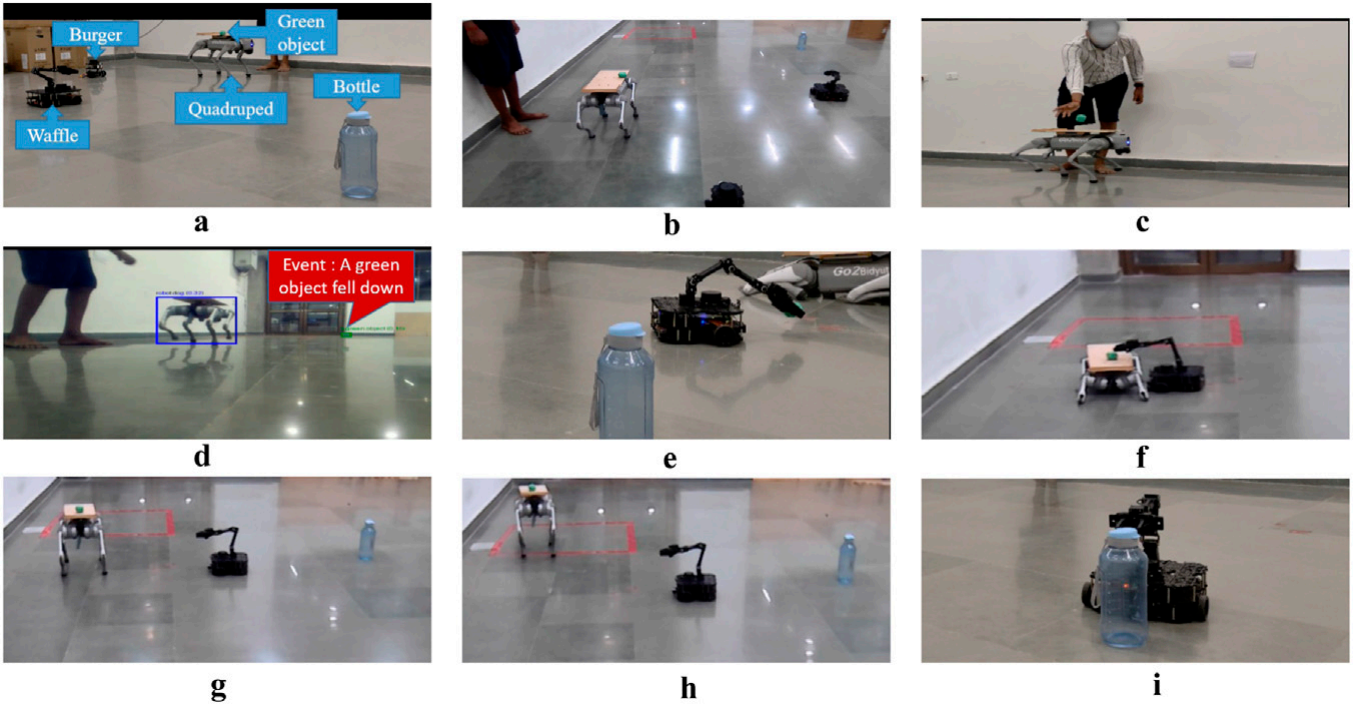
Demonstration of event-driven replanning, incomplete task resumption, and emergence of cooperation where robots assist one another. **(a)** Environment with quadruped carrying green object, Waffle mobile manipulator, Burger mobile robot and a Bottle. **(b)** Waffle moves toward the bottle; quadruped heads to its goal with the green object. **(c)** Human throws the green object off the quadruped’s back. **(d)** Burger detects the fall and raises an event; task manager replans. **(e)** Waffle picks up the green object. **(f)** Quadruped sits; Waffle places the object back. **(g)** Quadruped stands. **(h)** Quadruped goes to the goal. **(i)** Waffle resumes approaching the bottle.

As shown in Figures 7a,b, we deploy a Unitree Go2 quadruped, a mobile robot Turtlebot Burger, and a mobile manipulator Turtlebot Waffle-pi with an OpenManipulator-X arm (Waffle). The objective is to test whether independently tasked robots can recover collaboratively when a disruptive event occurs. The user instructs Waffle to approach a bottle (local LLM composes executable Python from its function library, e.g., find(object) and reach(object)), and the Go2 to carry a green object kept on its back to (3, 0).

Midway, the green object is deliberately knocked off the Go2 (Figure 7c). CoMuRoS performs (i) As shown in Figure 7d, Burger robot detects the green object and classifies it as relevant, (ii) triggers event-driven replanning, and (iii) reallocates sequential roles while respecting physical constraints. From the scenario configuration, the task manager infers that Waffle’s manipulator lacks reach to place the object on a standing quadruped and therefore instructs the Go2 to sit before the handover (Figure 7f). After Waffle completes the pick-and-place, Go2 resumes delivery (Figure 7h) and Waffle automatically resumes its original task (Figure 7i). Since its status was marked INTERRUPTED in the original plan, the task manager explicitly included it in the replanned sequence, ensuring completion of all assigned tasks. Across 10 trials (using GPT 4o), the success rate was **9/10**; failure arose from incorrect replanning sequence. The single failure case occurred due to incorrect sequential reordering during replanning due to LLM hallucinations, where the quadruped was instructed to resume motion before the object was secured. This experiment showed that multi-robot cooperation emerged as a response to a disruptive event. In this experiment event detection was done using color based identification and localization of object and then using LLM for reasoning about relevance of event to the current tasks.

**FIGURE 8:**
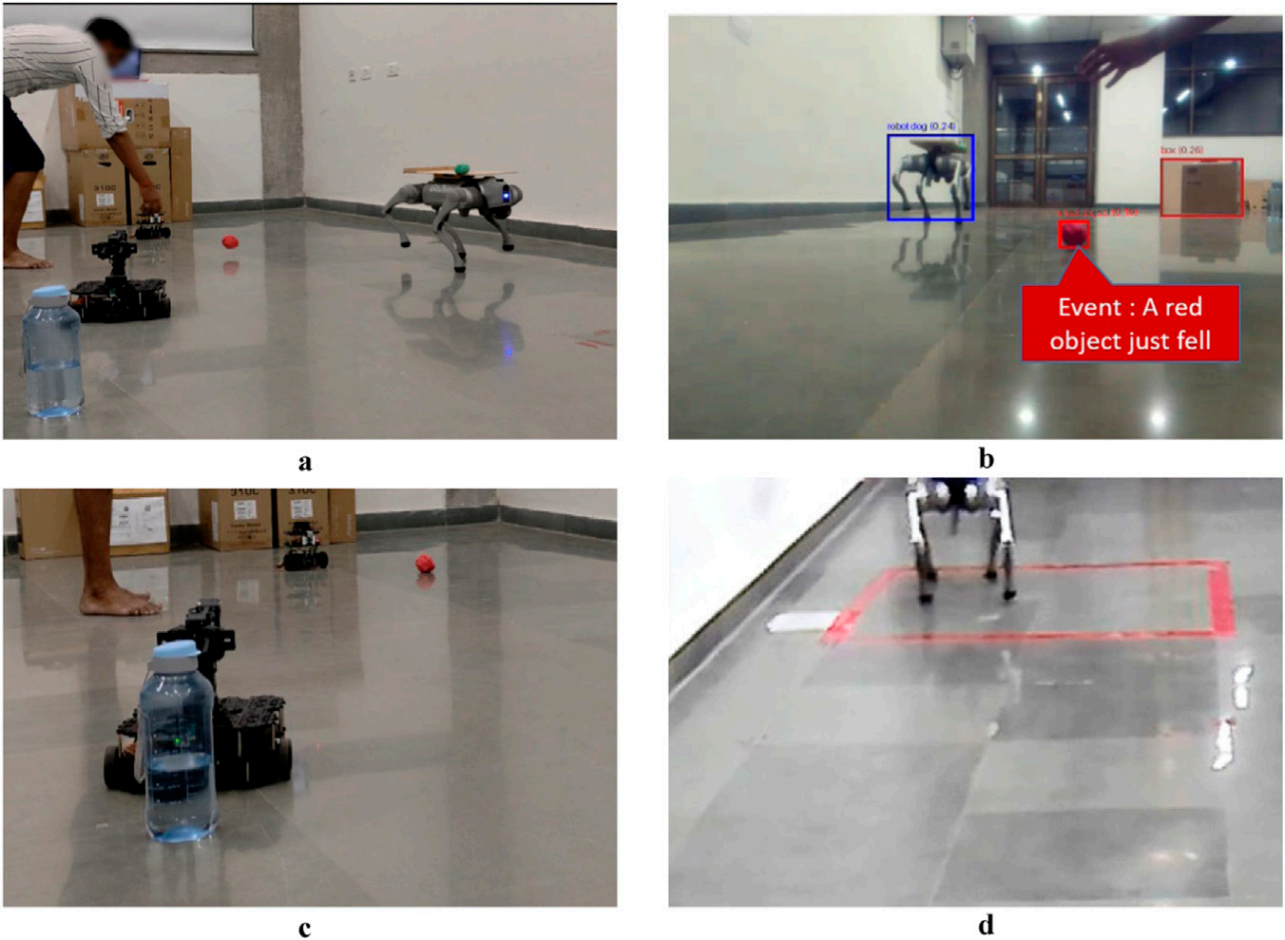
Demonstration of ignoring irrelevant events. **(a)** Human throws a red object in front of Burger robot. **(b)** Burger detects the red object fall event but ignores it as it is irrelevant to the task. **(c)** Waffle reaches the bottle. **(d)** Quadruped reaches its goal location.

A separate experiment demonstrates filtering of irrelevant events. When a red object is thrown in front of Burger (Figure 8a), the robot detects the object (Figure 8b) but classifies it as irrelevant, so no event is communicated to the task manager. Both Waffle and Go2 complete their assigned tasks without interruption (Figures 8c,d). This shows how decentralized event classification reduces unnecessary processing load on the task manager while keeping the system focused on relevant events only. This experiment had a success rate of 10/10.

#### 4.1.2 Human-formation collaboration

**FIGURE 9:**
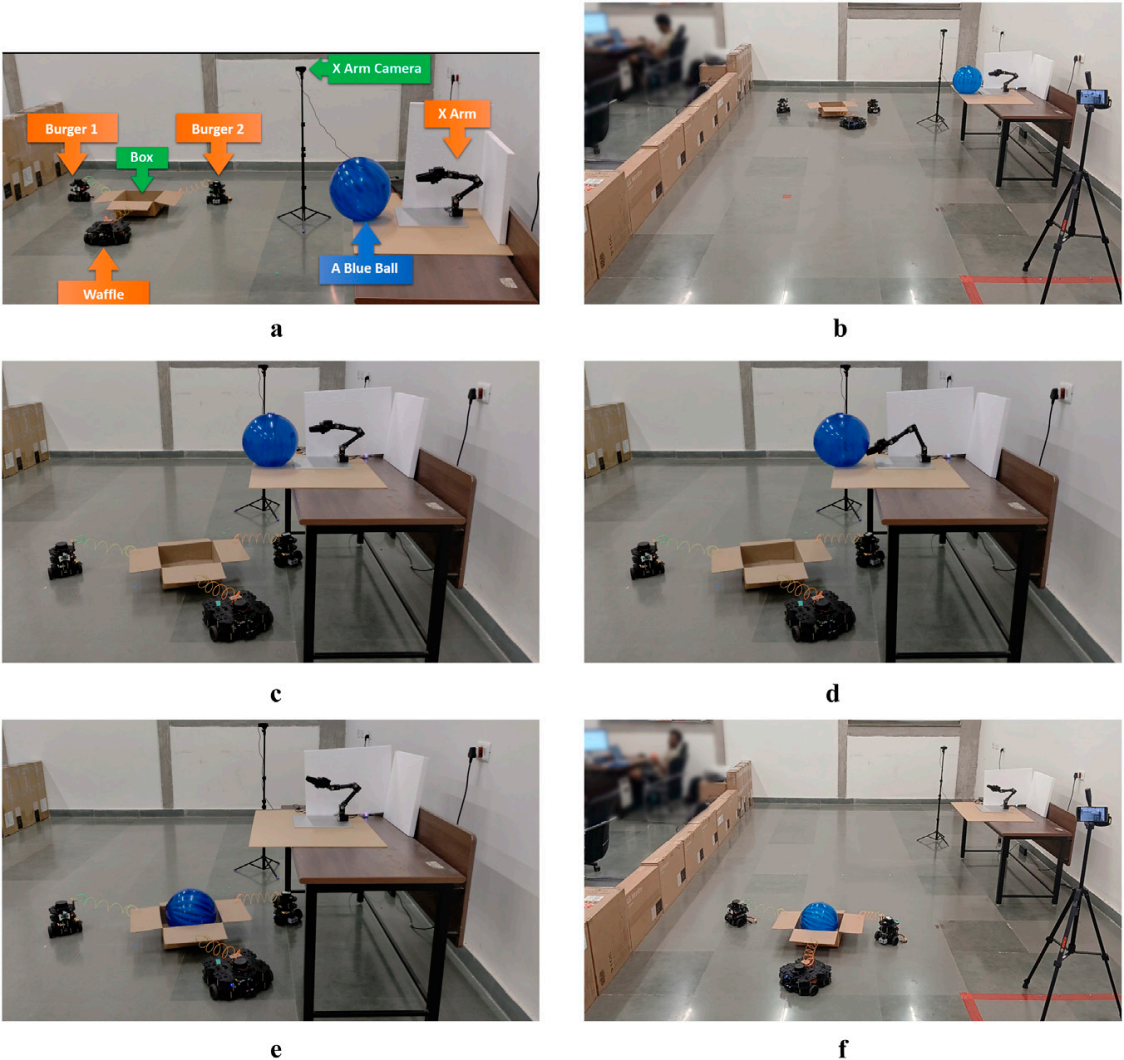
Demonstration of CoMuRoS performing a coordinated task: the X-arm and a mobile robot formation collaborate to transfer the ball from the table to the goal location (4,0). **(a)** Initial scene overview. **(b)** Formation moving to catch point. **(c)** X-arm pushing ball. **(d)** Ball pushed into box. **(e)** Ball inside box. **(f)** Robots deliver ball to (4,0).

**FIGURE 10:**
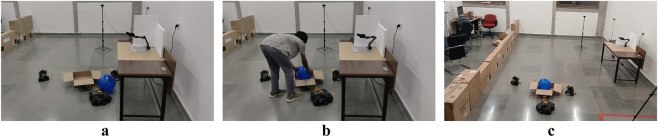
Demonstration of human-multi-robot collaboration resulting from failure. **(a)** Ball falls outside the box instead of inside. **(b)** On request from the task manager, the human puts the ball inside the box. **(c)** Formation carries the ball towards the goal.

Figure 9a shows the scene overview of this experiment. A formation of three mobile robots (One Waffle and two Burger robots) can carry a box. There is a table with a blue ball and an OpenManipulator-X arm (X-arm). The user instructs the multi-robot system to “Deliver the blue ball to (4,0)”. The scenario configuration file has the description of the scene and catch point coordinates where the formation can collect the ball from the X-arm. The task manager classifies the task as sequential and coordinated, generating a plan shown in the chat interface Figure 4. According to this plan and as shown in Figures 9b–e formation of mobile robots carries the box to the catch point, and the X-arm pushes the ball off the table. The X-arm camera detects whether the ball has fallen into the box successfully. If the ball falls into the box, the X-arms task status is marked COMPLETED and the formation delivers the box to the goal position successfully. This experiment had a success rate of 8/8. Figure 10 shows what happens if the X-arm task fails, i. e., after pushing, the ball falls outside the box. The X-arm’s camera detects the event, notifies it to the task manager and to the human chat interface, as shown in green in Figure 4. This event, being relevant to the task, triggers replanning. Since none of the robots has the ability to keep the ball back inside the box, the task manager assigns the task to the user as shown in Figure 4.

User places the ball into the box and then the formation carries the ball to goal position successfully, as shown in Figure 10. The events of the ball falling inside the box or outside, or the placement of the ball inside the box, were detected using the VLM of GPT 4.1 for Visual Question Answering (VQA). This experiment shows the ability of CoMuRoS architecture to enable human-robot collaboration, the ability to identify if a task is beyond robots’ capabilities and in that case, ask for help. This experiment has a success rate of **5/5**. In order for the ball to fall outside the box, the table position was slightly modified compared to the previous experiment. Human collaboration can significantly improve the robustness of task completions in real settings.

### 4.2 Simulation

In order to show relevance of CoMuRoS to diverse and real problems, simulations were performed in Gazebo environment for two scenarios Hospital and Disaster Relief.

#### 4.2.1 Task allocation in disaster relief scenario

**FIGURE 11:**
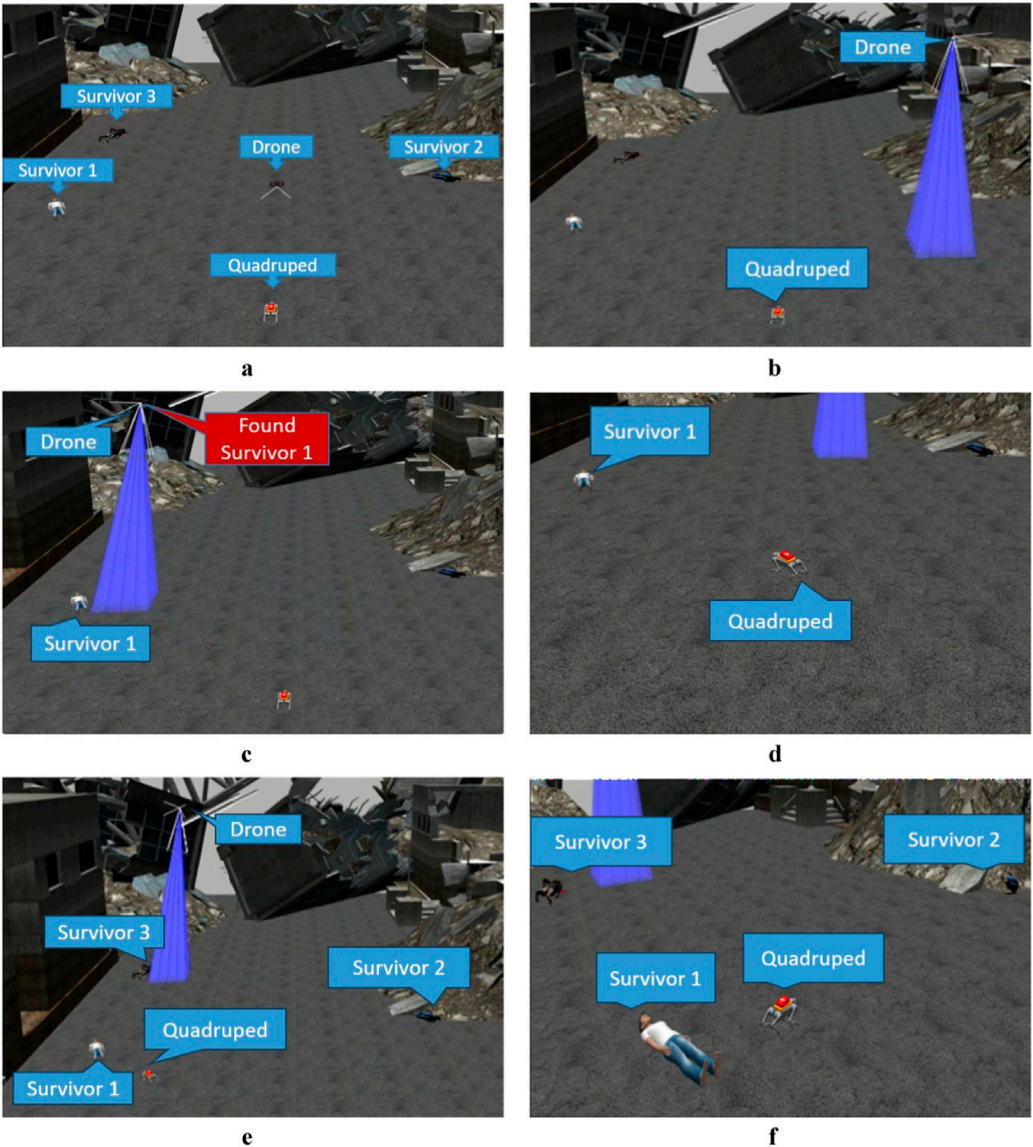
Demonstration of task allocation, classification, and replanning in a disaster relief scenario. **(a)** Overview of scene. **(b)** Drone starts surveillance. **(c)** Drone finds a survivor and reports coordinates. **(d)** Quadruped moves to survivor 1 with first aid kit. **(e)** Drone finds survivors 2 and 3 and reports coordinates. **(f)** Quadruped reaches survivor 1 then moves to next.

In the disaster relief scenario (Figure 11), a Go2 quadruped and a drone are tasked with finding survivors and delivering first aid kits. The user command, “Find all survivors and deliver first aid kits to them,” is classified as an independent task: the drone searches while the quadruped delivers. When the drone detects a survivor, it raises a relevant event, triggering replanning. The task manager provides the survivor’s coordinates to the quadruped while the drone continues scanning for new survivors.

#### 4.2.2 Understanding human intentions: multirobot food delivery

**FIGURE 12:**
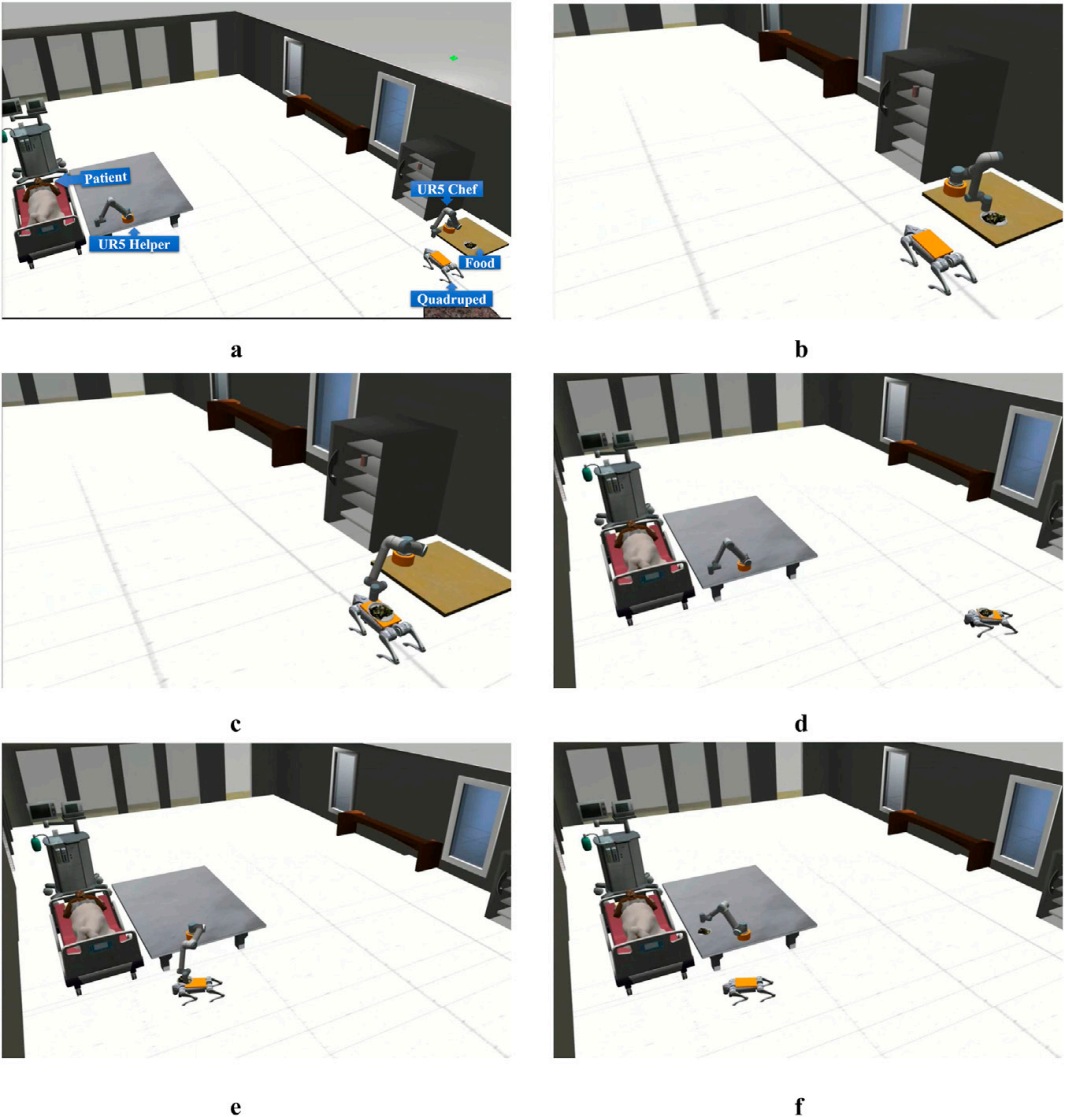
Hospital scenario: interpreting the human prompt “I am hungry” and coordinating UR5 arms with a quadruped to deliver food. **(a)** Initial scene overview. **(b)** Patient says: “I am hungry”. **(c)** UR5 chef places plate on quadruped. **(d)** Quadruped moves toward patient. **(e)** UR5 helper takes plate. **(f)** UR5 places plate on table.

**FIGURE 13:**
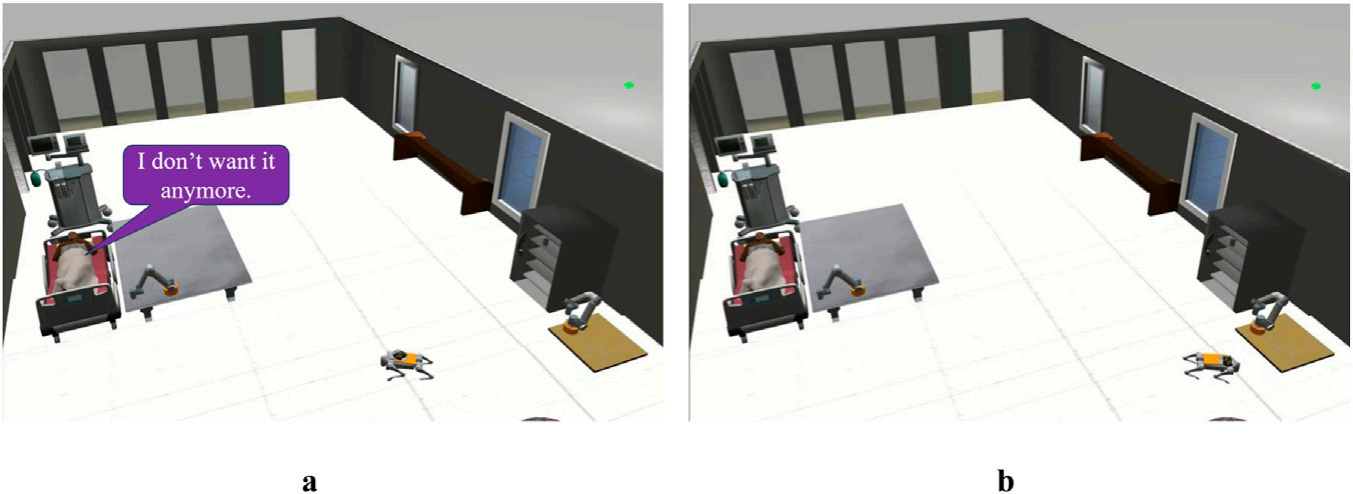
Hospital scenario: demonstration of anytime human interruption, with the quadruped adapting its plan based on updated intent. **(a)** Patient refuses food. **(b)** Quadruped returns food to table.

In the hospital scenario (Figure 12a), a Unitree Go2 quadruped and two UR5 arms collaborate to deliver food after the user says, “I am hungry.” The chef arm loads the plate, the quadruped carries it, and the helper arm serves it (Figures 12b–f). CoMuRoS also supports anytime human input: in Figure 13, when the user interrupts with “I do not want it anymore,” this is classified as a relevant event, and replanning directs the quadruped to return the food. This demonstrates the system’s ability to interpret indirect human intent as none of the actions of pick, place and move was not in user prompt. Also this experiment show ability of system to incorporate anytime user feedback through the human interface and respond to it by replanning if required.

**FIGURE 14:**
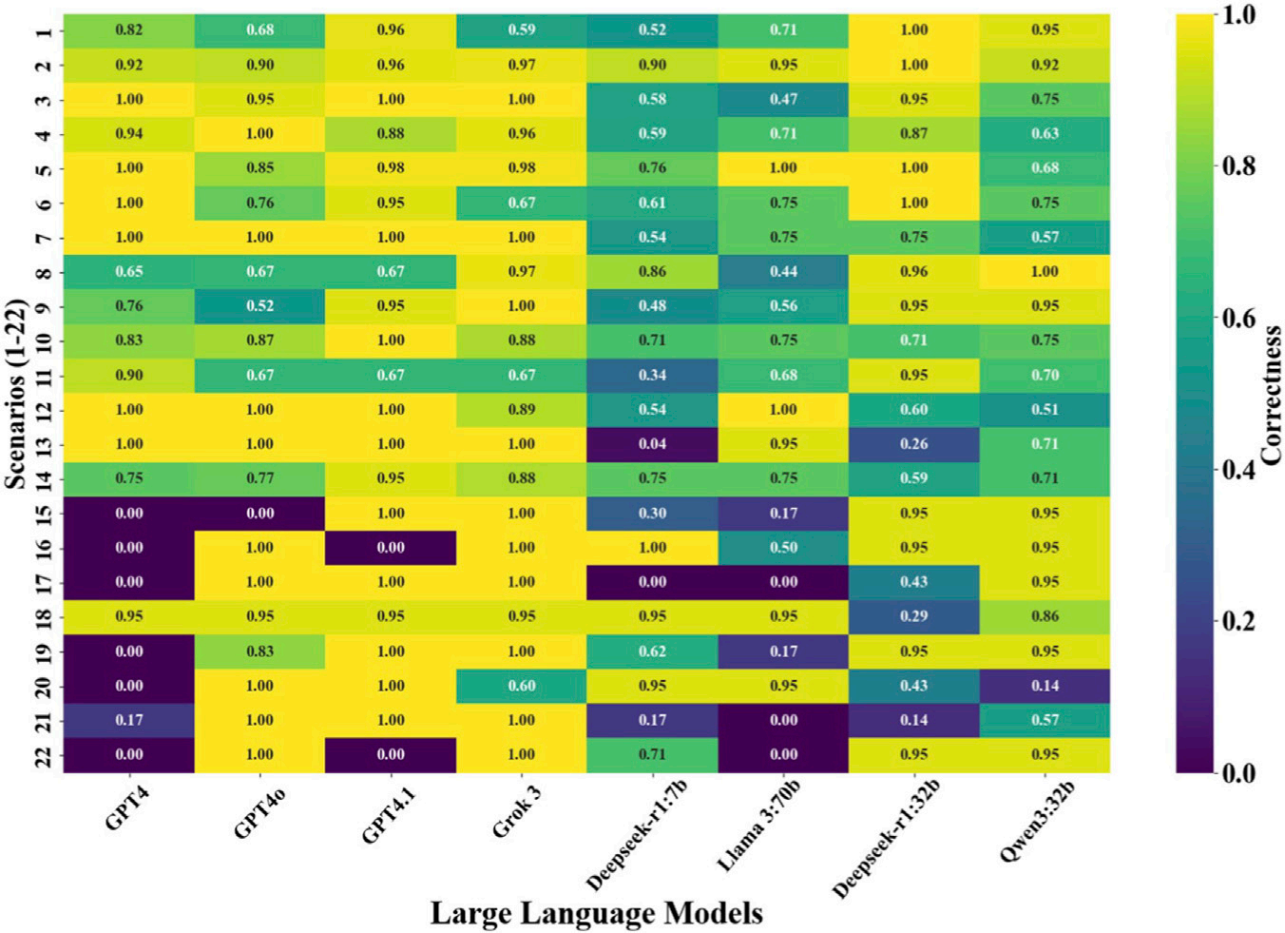
Correctness scores across scenarios(1-22) across different LLMs(GPT 4, GPT 4o, GPT 4.1, Grok 3, DeepSeek-r1:7b, Llama 3:70b, DeepSeek-r1:32b and Qwen3:32b). The 22 scenarios are 1. Reforestation, 2. Airport Baggage Handling, 3. Asteroid Mining, 4. Beach Cleanup, 5. Border Patrol, 6. Building Construction, 7. Agriculture, 8. Gardening, 9. Road Cleaning, 10. Hospital, 11. Home Clutter Sorting, 12. Mine Detection, 13. Office, 14. Classroom, 15. Shopfloor, 16. Car Repair, 17. Rubble Clearance, 18. Fire-fighting, 19. Solar Panel Installation, 20. Rescue, 21. Interior Services, 22. Boat Hauling.

## 5 Generalization results across tasks, scenarios and LLMs

### 5.1 Performance across the textual task planning dataset

CoMuRoS architecture’s high-level planning capability was evaluated using a manually curated textual benchmark dataset for generalization across 22 scenarios, with 54 tasks evaluated by eight different LLMs (APIs and local). Each task is evaluated five times.

**FIGURE 15:**
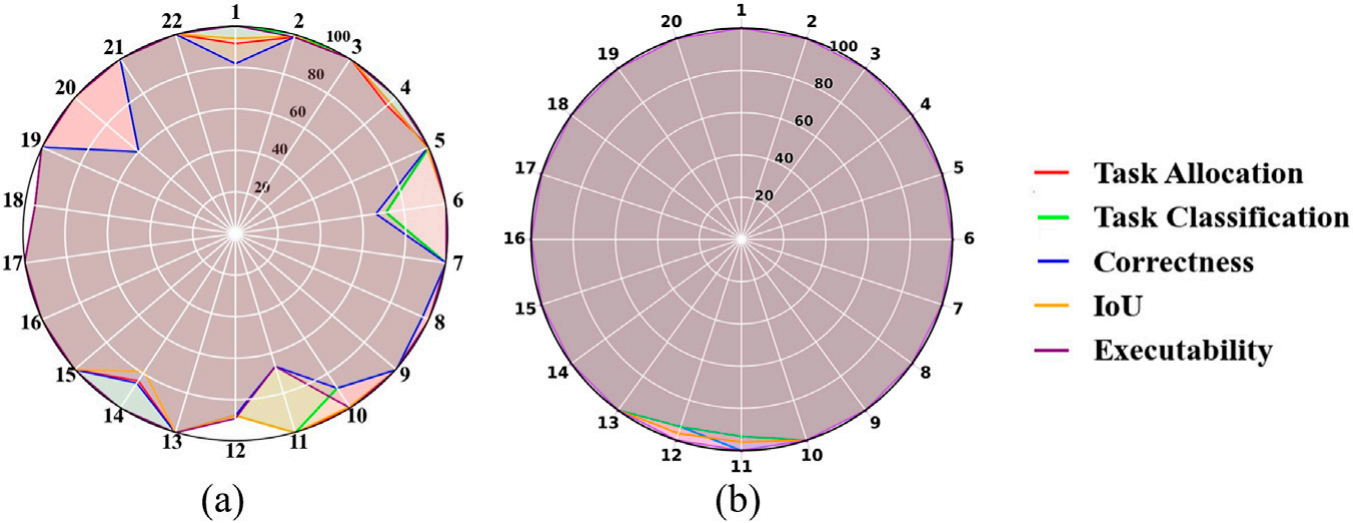
**(a)** Per-metric performance across 22 scenarios(mentioned in Figure 14) for Grok 3. Each axis is a scenario, with values showing average metric scores (0-1) across all tasks and iterations. **(b)** Evaluation for replanning in different tasks for Grok 3 across 20 scenarios. The 20 scenarios are 1) Railway Station, 2) School Robots, 3) Movie Theaters, 4) Space Station, 5) Shopping Mall, 6) Data Center, 7) Power Plant, 8) Warehouse, 9) Cruise Ship, 10) Underwater Lab, 11) Crowd Management, 12) Theme Park, 13) Museum, 14) University Campus, 15) Robotics Lab, 16) Hotel, 17) Zoo, 18) Pharmacy Shop, 19) Water Park, 20) Subway Station.

As seen in Figure 14 CoMuRoS performs better using Grok 3 than other LLMs. CoMuRoS has an average correctness of 0.91 while using Grok 3 (averaged across 22 scenarios having in total 54 tasks, and each task was planned 5 times). To validate the objectivity of the correctness metric, a second independent evaluator scored 160 task manager LLM outputs (Grok 3 and DeepSeek-r1:32b) corresponding to randomly selected task-scenario pairs from the dataset, yielding Cohen’s κ=0.751 , indicating substantial agreement (Landis and Koch, 1977). The average values for Grok 3 are: TA =0.96±0.024 , TC =0.963±0.004 , Correctness =0.91±0.053 , IoU =0.987±0.015 , Executability =0.96±0.016 Figure 15a shows CoMuRoS performance on these metrics across all scenarios.

**FIGURE 16:**
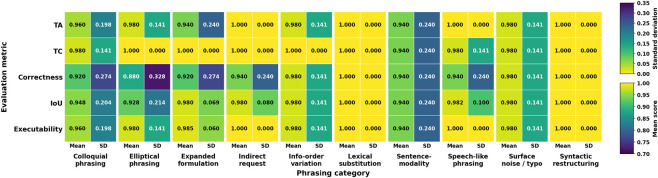
Sensitivity of the Task Manager under different phrasing variations. Each phrasing category was evaluated using five metrics: Task Allocation (TA), Task Classification (TC), Correctness, IoU, and Executability. For each category, the mean score and standard deviation are shown as alternating columns. Higher mean scores indicate better task-manager performance, while lower standard deviation indicates more consistent behavior across tasks. Mean TA = 0.976 ± 0.12, mean TC = 0.99 ± 0.06, mean Correctness = 0.96 ± 0.19, mean IoU = 0.97 ± 0.13, mean Executability = 0.98 ± 0.10 across all phrasing categories. Elliptical phrasing showed minimum mean of 0.88 ± 0.328 in Correctness metric.

### 5.2 Performance on textual replanning dataset

A second dataset of 20 scenarios (3 tasks each) evaluates replanning using the same metrics. For each scenario, the dataset provides an initial user command and an event description. The task manager generates an initial plan, then replans after the event. The new plan is evaluated on the same metrics only when the initial plan is correct and the event is relevant. Each task is tested 5 times. As shown in Figure 15b, CoMuRoS achieves average TA =1.00±0.00 , TC =0.969±0.017 , Correctness =0.948±0.034 , IoU =0.973±0.018 , Executability =1.00±0.00 , across all 20 scenarios with 5 tasks each tested 5 times with Grok 3. The replanning benchmark is intended as a complementary evaluation alongside the hardware experiments (Section 4), which provide the primary evidence of runtime event-driven replanning on physical robots (9/10, 8/8, 5/5). The textual replanning benchmark evaluates the task manager’s replanning logic across diverse scenario types and event descriptions at scale, which would be impractical to replicate entirely in hardware.

### 5.3 Phrasing-robustness evaluation

To evaluate whether the Task Manager is robust to meaning-preserving variations in user instructions, we constructed a phrasing-robustness benchmark in which each task intent was rewritten using ten linguistic categories: colloquial phrasing, elliptical phrasing, expanded formulation, indirect request, information-order variation, lexical substitution, sentence-modality variation, speech-like phrasing, surface-noise/typo variation, and syntactic restructuring. These categories were chosen to cover both formal paraphrase phenomena and practical variations expected in human–robot interaction. Lexical substitution changes key words while preserving the task meaning; for example, the airport baggage-handling task was expressed as “Transfer the luggage from the terminal belt into the aircraft” instead of the normal prompt “Pick up the bag from the terminal belt and load it into the airplane.” Syntactic restructuring changes grammatical form while preserving the underlying semantic roles, as in “The baggage from the conveyor belt should be loaded into the airplane.” Information-order variation changes the order in which the source, object, and destination are mentioned, as in “From the arrival belt, move the bags into the aircraft cargo hold.” Sentence-modality variation changes the directive form from an imperative to a request or question, as in “Could you load the baggage from the terminal system into the plane?” Elliptical phrasing removes grammatical detail while leaving the intent recoverable, as in “Baggage belt to aircraft cargo.” Expanded formulation adds contextual or explanatory wording, as in “The boarding process begins shortly, so move the checked baggage from the conveyor line into the assigned airplane.” These six categories are motivated by established paraphrase typologies that identify lexical, syntactic, discourse-order, modality, ellipsis, and addition/deletion-based reformulations as common meaning-preserving transformations (Vila et al., 2014). Indirect request phrasing expresses the task pragmatically rather than as an explicit command, for example, “The luggage still has not been loaded onto the aircraft,” and is included because task-oriented dialogue often contains implied user intents rather than direct instructions (Mannekote et al., 2025). Colloquial phrasing captures informal user commands, such as “Get those bags onto the plane before departure,” while speech-like phrasing captures conversational ordering, hesitation, or spoken-style sequencing, such as “Right, collect the baggage from the belt first—then load it into the cargo section.” These categories are motivated by task-oriented dialogue robustness studies, which identify language variety and speech characteristics as important sources of brittleness in language understanding systems (Liu et al., 2021). Finally, surface-noise/typo variation introduces realistic input corruption, such as “trnsfr baggage belt 2 aircraft,” reflecting misspellings, abbreviations, punctuation variation, and other real-world noise patterns known to affect intent classification and slot labeling in goal-oriented dialogue systems (Sengupta et al., 2021). As shown in Figure 16, for each phrasing category, we evaluated ten task intents using Grok-3. Each task-phrasing pair was executed using Grok3 over five independent generations, and the outputs were evaluated using five metrics: Task Allocation (TA), Task Classification (TC), Correctness, IoU, and Executability. For each task-phrasing pair, the five repeated runs were first averaged; the reported mean and standard deviation were then computed across the ten task intents within each phrasing category.

The average task manager call latency was 1.83 s ± 0.51 s for GPT-4o, 3.71 s ± 1.93 s for Grok-3, 4.01 s ± 1.07 s for DeepSeek-r1:7b (local deployment on RTX 4090) and 71.0 s ± 17.0 s for DeepSeek-r1:32b (local deployment on RTX 4090) across 20 benchmark tasks. Latency scales sub-linearly with team size as the task manager is queried once per planning cycle and robot-level LLMs are queried in parallel or sequentially depending on task classification and task status. More detailed scalability analysis will be part of future work.

## 6 Conclusion

This work presents a systems-level hierarchical multi-robot architecture, CoMuRoS, integrating Large Language Models for planning, with contributions at the architecture and systems integration level: a classification-guided hierarchical task dispatch mechanism, runtime event-driven replanning validated on physical heterogeneous robots with emergent multi-robot cooperation, and a modular scenario-agnostic design enabling zero-shot generalization of the task planning context across diverse applications without modifying the core planning logic. The task manager redistributes tasks in response to events, inspired by human teams where members can signal issues and managers adapt roles. Hardware trials on heterogeneous robots and simulations show high success rates. The architecture is both modular and generalizable: keeping the static prompt and core task manager logic unchanged, and modifying only the scenario configuration file, gave consistent success across textual datasets, simulations, and hardware experiments. To the best of our knowledge, this is one of the first demonstrations of runtime event-driven replanning and emergent multi-robot cooperation in LLM-based multi-robot systems (Table 1), where task failures trigger dynamic reallocation and robots autonomously assist one another to ensure task completion. CoMuRoS is designed for open-world deployment scenarios where the space of possible tasks, events, robot configurations, and environmental conditions cannot be strictly pre-enumerated. New objects, locations, rules, and constraints can always be naturally integrated by describing them in the scenario configuration file in natural language, without requiring modification of formal models or dependency graphs. This trades formal correctness guarantees, offered by systems with explicit representations such as DAGs, behavior trees, or PDDL, for open-world adaptability and zero-shot generalization to new scenarios and robot configurations without modifying the core planning logic. Future advances in foundation models and LLMs promise even more flexible, human-like collaboration.

### 6.1 Limitations and future directions

CoMuRoS operates under several assumptions that should be explicitly acknowledged: robot capabilities and morphological constraints must be manually specified in the scenario configuration file; low-level skill functions must be pre-implemented per robot type; and application-specific rules must be domain-engineered by the user. The degree of explicit environment specification additionally depends on robot sensing capabilities: when robots have onboard sensing and object-finding capabilities, natural language descriptions in the scenario configuration are sufficient and object locations need not be explicitly specified, as demonstrated in Hardware Experiment 1 (Section 4.1.1), where the green object was located using onboard color-based sensing without explicit coordinate specification. Where robots lack such sensing capabilities, important locations can be provided explicitly in the scenario configuration file, as in Hardware Experiment 2 (Section 4.1.2), where the catch point coordinates are specified explicitly, and in the hospital simulation, where delivery coordinates are provided. CoMuRoS thus supports a spectrum from fully natural-language-described to fully coordinate-specified environments, determined by the sensing capabilities of the deployed robot team. Replanning currently ensures task completion after relevant events; future directions include reasoning about unrecoverable situations and pursuing partial completion to minimize loss. Further work will explore utilizing a map of the environment as context. Developing advanced context management for token efficiency is an important future addition. This work uses a naive approach of processing all video chunks of fixed size for event detection, making reliable detection expensive when using APIs, while offline LLMs and VLMs are not yet sufficiently accurate for general tasks and also introduce higher inference latency compared to API-based models. Choosing a fixed video chunk size can potentially lead to loss of information for very slow or fast events; hence future work will focus on developing better event detection algorithms with adaptive chunking strategies. The relationship between scenario configuration length, task manager prompt size, inference latency, and planning correctness is an important direction for future study. The current monolithic task manager prompt may also be decomposed into a network of specialized agents running in parallel to reduce latency while maintaining correctness. Scalability of CoMuRoS to large robot teams is an open and important research question. While CoMuRoS has been demonstrated with up to 4 heterogeneous robots in hardware experiments, and Chen et al. (2024) suggest that a two-level hybrid hierarchical architecture scales better than fully centralized or decentralized approaches, it remains to be assessed whether the current two-level hierarchy is sufficient for spatially distributed deployments involving hundreds or thousands of robots, such as an entire factory floor. Such settings may require a tree-like multi-level planning hierarchy, where multiple layers of task managers coordinate specialized sub-teams at different levels of abstraction, and the optimal depth and breadth of such a hierarchy for a given deployment scale remains an important open question for future investigation. Furthermore, CoMuRoS currently targets task completion rather than optimal execution; evaluating and optimizing for plan efficiency, resource utilization, and minimizing total task completion time represent important future directions. Human-robot collaboration in CoMuRoS is demonstrated through the hardware interface including task allocation to humans and human-in-the-loop recovery; a formal user study evaluating response time, cognitive load, trust, and interaction quality in human-robot teaming scenarios remains as future work. An interesting future direction is the development of persistent memory and user preference modeling, where the task manager continuously adapts from earlier mistakes, failures, and user interactions, building a long-term context that improves planning quality over repeated deployments rather than treating each session independently. Several ablation studies would be interesting future directions, including evaluating the impact of different prompt structures on task classification accuracy, comparing LLM-generated code execution against scripted skill execution, and assessing the effect of varying scenario configuration detail on task allocation correctness; these were not conducted in the current work due to time constraints. The absence of head-to-head runtime comparison against prior systems is acknowledged as a limitation of the current evaluation, further complicated by the fundamentally different input-output contracts between CoMuRoS and prior systems that rely on explicit formal task representations. Standardized benchmarks for LLM-based multi-robot systems that include event-driven replanning capability would enable more rigorous future comparisons and are identified as an important community need. A natural future extension of this work is to support asynchronous task execution, where robots can initiate a task, temporarily leave it to perform another task, and later resume it, thereby enhancing system flexibility and efficiency.

## Data Availability

The datasets presented in this study can be found in online repositories. The names of the repository/repositories and accession number(s) can be found below: comuros. github.io.
