# Supplementary material for: CoMuRoS - An LLM-based generalizable hierarchical task planning and execution framework for heterogeneous robot teams with event-driven re-planning
Source: Front Robot AI. 2026 Aug 4;13:1843313. doi: 10.3389/frobt.2026.1843313 (PMC13482247; doi:10.3389/frobt.2026.1843313)
Supplement: Supplementary file 1 [file Supplementaryfile1.pdf]

Supplementary Appendix A: Skill Libraries for Hardware and Simulation Experiments

Each robot node maintains a compact dictionary of skill functions with associated descriptions and example usage. Object locations and goal positions are provided via the scenario configuration file as explicit system inputs. All skill libraries are also available at CoMuRoS.github.io.

A.1 Hardware Experiment 1: Multi-Robot Collaboration

Go2 Quadruped (3 skills):

- `goto(goal)`: Navigate to any location.
- `sit_down()`: Command the robot to sit.
- `stand_up()`: Command the robot to stand.
  These behaviors are called internally inside the skill functions using the Unitree SDK2 Python interface (unitreerobotics, 2024).

Waffle Mobile Manipulator (4 skills):

- `goto(object)`: Navigate to any location.
- `find(object)`: Locate any object in the environment.
- `pick(object)`: Pick up a named object.
- `place(object)`: Place a named object.
  Object detection and navigation are performed using Grounding DINO (Liu et al., 2023). Both `find` and `goto` use vision-based PD control; code is available on the project website. The `goto` function additionally uses a front-mounted ultrasonic sensor to estimate the linear distance error to the object, while Grounding DINO bounding boxes are used to estimate the lateral displacement error. The `pick` and `place` functions are implemented using MoveIt (Coleman et al., 2014).

A.2 Hardware Experiment 2: Human-Formation Collaboration

Formation Team (1 skill):

- `goto(goal)`: Navigate the formation to any location.
  The formation-control function for the Burger robots is implemented using ChoiRbot (Testa et al., 2021).

X-Arm Manipulator (1 skill):

- `push()`: Push the object in front of the robot.
  The `push` function is implemented using MoveIt (Coleman et al., 2014).

A.3 Simulation: Hospital Scenario

Go2 Quadruped (5 skills):

- `goto(goal)`: Navigate to any location.
- `find(object)`: Locate any object in the environment.
- `sit_down()`: Command the robot to sit.
- `stand_up()`: Command the robot to stand.

- `stop()`: Stop the robot immediately.
  In simulation, the Go2 quadruped skills are implemented using the CHAMP controller provided by Jain (2024); this controller is based on the hierarchical locomotion control framework of Lee (2013).

UR5 Arm (2 skills):

- `pick(object)`: Pick up a named object.
- `place(object)`: Place a named object.
  The `pick` and `place` functions are implemented using MoveIt (Coleman et al., 2014), with object attachment handled through the IFRA LinkAttacher Gazebo–ROS 2 plugin (IFRA-Cranfield, 2024).

A.4 Simulation: Disaster Relief Scenario

Go2 Quadruped (5 skills): Same as hospital scenario above (Section A.3).
Drone (3 skills):

- `start_surveillance()`: Begin aerial surveillance of the area.
- `land()`: Land the drone.
- `take_off()`: Take off the drone.
  The quadruped skills use the same Unitree Go2 ROS 2 interface and controller setup as in the hospital scenario (Jain, 2024; Lee, 2013). The drone skills are implemented using the SJTU drone ROS/ROS 2 Gazebo simulator (No, 2025).

References

David Coleman, Ioan Sucan, Sachin Chitta, and Nikolaus Correll. Reducing the barrier to entry of complex robotic software: a moveit! case study. *arXiv preprint arXiv:1404.3785*, 2014.

IFRA-Cranfield. Ifra_linkattacher - gazebo-ros2 plugin. https://github.com/IFRA-Cranfield/IFRA_LinkAttacher, 2024. Accessed: 2026-05-26.

Anuj Jain. unitree-go2-ros2. https://github.com/anujjain-dev/unitree-go2-ros2, 2024. GitHub repository, accessed: 2026-05-26.

Jongwoo Lee. *Hierarchical controller for highly dynamic locomotion utilizing pattern modulation and impedance control: Implementation on the MIT Cheetah robot.* PhD thesis, Massachusetts Institute of Technology, 2013.

Shilong Liu, Zhaoyang Zeng, Tianhe Ren, Feng Li, Hao Zhang, Jie Yang, Chunyuan Li, Jianwei Yang, Hang Su, Jun Zhu, et al. Grounding dino: Marrying dino with grounded pre-training for open-set object detection. *arXiv preprint arXiv:2303.05499*, 2023.

Georg No. sjtu_drone: Ros/ros2 gazebo quadcopter simulator. https://github.com/NovoG93/sjtu_drone, 2025. GitHub repository, accessed: 2026-05-26.

Andrea Testa, Andrea Camisa, and Giuseppe Notarstefano. Choirbot: A ros 2 toolbox for cooperative robotics. *IEEE Robotics and Automation Letters*, 6(2):2714–2720, 2021.

unitreerobotics. unitree_sdk2_python. https://github.com/unitreerobotics/unitree_sdk2_python, 2024. GitHub repository, accessed: 2026-05-26.
